# Predicting Turns in Proteins with a Unified Model

**DOI:** 10.1371/journal.pone.0048389

**Published:** 2012-11-07

**Authors:** Qi Song, Tonghua Li, Peisheng Cong, Jiangming Sun, Dapeng Li, Shengnan Tang

**Affiliations:** Department of Chemistry, Tongji University, Shanghai, China; CSIR-Institute of Microbial Technology, India

## Abstract

**Motivation:**

Turns are a critical element of the structure of a protein; turns play a crucial role in loops, folds, and interactions. Current prediction methods are well developed for the prediction of individual turn types, including α-turn, β-turn, and γ-turn, etc. However, for further protein structure and function prediction it is necessary to develop a uniform model that can accurately predict all types of turns simultaneously.

**Results:**

In this study, we present a novel approach, TurnP, which offers the ability to investigate all the turns in a protein based on a unified model. The main characteristics of TurnP are: (i) using newly exploited features of structural evolution information (secondary structure and shape string of protein) based on structure homologies, (ii) considering all types of turns in a unified model, and (iii) practical capability of accurate prediction of all turns simultaneously for a query. TurnP utilizes predicted secondary structures and predicted shape strings, both of which have greater accuracy, based on innovative technologies which were both developed by our group. Then, sequence and structural evolution features, which are profile of sequence, profile of secondary structures and profile of shape strings are generated by sequence and structure alignment. When TurnP was validated on a non-redundant dataset (4,107 entries) by five-fold cross-validation, we achieved an accuracy of 88.8% and a sensitivity of 71.8%, which exceeded the most state-of-the-art predictors of certain type of turn. Newly determined sequences, the EVA and CASP9 datasets were used as independent tests and the results we achieved were outstanding for turn predictions and confirmed the good performance of TurnP for practical applications.

## Introduction

It is widely acknowledged that the function of a protein is determined by its structure to a certain extent. In the field of protein research, the gap between sequences and proteins with known structures or functions is very large and it is growing at an exponential rate. To narrow the gap, numerous protein structure prediction methods have been developed.

Turn is a special secondary structure in proteins, which plays a crucial role in loops, folds, and interactions. Turns and loops are all in the flexible region of a protein, a short hairpin loop can also be called a simple turn [1]. The accurate prediction of protein turns is undoubtedly useful for loop identification. In protein folding, turns play a critical part by bringing together regular secondary structure elements. Turn often defines the three-dimensional arrangement of other secondary structures, such as α-helices or β-sheets, thereby determining the overall architecture of a protein domain [2]. Furthermore, some turns typically occur on the exposed surface of proteins and, hence, are likely to be involved in the molecular recognition of the protein environment or in interactions between peptide substrates and receptors [3].

Different from α-helices and β-sheets, turn is an irregular secondary structure; turn is the site where a peptide chain reverses its overall direction. Turns typically involve a hydrogen bond between the first and the last residue or have a distance between the first and last residue of less than 7 Å. Turns can be classified as δ, γ, β, α, and π types, which are formed by 2, 3, 4, 5, and 6 residues, respectively [2], [3].

Several excellent methods have been developed to predict each turn category. β-turn is the most commonly found turn, and it can be classified into nine types: I, II, VIII, I’, II’, VIa1, VIa2, VIb, and IV [4]. One of the best methods for the prediction of β-turns [5] achieved Accuracy of 87.2% on the BT436 dataset, using predicted shape strings as a new variable and a two-layer support vector machine (SVM) model. Except machine learning method [6], [7], [8], [9], statistical methods also been proposed [10], [11]. γ-turn is the second most characterized tight turn, which involves three amino acid residues and a hydrogen bond between the backbone CO_(i)_ and the backbone NH_(i+2)_. The problems of γ-turn prediction can be divided into two categories: prediction of γ-turn types [12] and prediction of γ-turn/non-γ-turn [13], [14], [15]. The method proposed by our group [15], which utilized G-means metrics as the optimal criterion for SVM and predicted shape string as a new variable. α-turn is always present on the exposed surface of the protein and contains specific information for the molecular recognition process. Both of SVM [16] and Artificial neural network [17] were launched to predict α-turn using PSI-Blast Profiles. The SVM-based classifier could also be used to predict π-turn, which is the longest tight turn, with PSSMs (Position Specific Scoring Matrixs) and predicted secondary structure as inputs [18]. However, there are few methods that developed for the prediction of all the different types of turns in a protein. Recently, Michael Meissner [2] provided a uniform classification for all turn families and predicted turn and non-turn by using self-organizing map (SOM) and SVM. We recognize that as a regular shape state, turn may have some intrinsic characteristics that affect the scope of the protein structure. Therefore, it is useful to predict all turns in a protein based on a unified model to perfect secondary structure prediction and to prepare tertiary structure prediction.

In this study, we present a novel approach, TurnP, for the prediction of global turns in a protein. We have developed a strategy to predict turns based on sequence and structural evolution information, which were PSSM (produced by PSI-BLAST) [19], predicted protein secondary structure information (secondary structure and Structural Position-Specific Scoring Matrix (SPSSM)) [20], and predicted protein shape string information (shape string and shape string profile) [21]; the method used the Conditional Random Field (CRF) [22] as an algorithm. After training with a non-redundant dataset (4,107 entries), TurnP was tested on a newly determined dataset, an EVA set and the CASP9 dataset. The results we obtained indicated that TurnP showed impressive performance in the field of turn prediction.

## Materials and Methods

### 1 Datasets

Two major datasets were constructed for the method formation: a training set Train_0925 and a test set Test_1025. Train_0925 contains 4,107 chains, which were derived from sequences in the Protein Data Bank (PDB) [23] released before 2010 that were determined by X-ray diffraction with a resolution better than 2.0 Å. Using PISCES [24], we determined that there are no two chains that have more than 25% sequence identity. The list of PDB ID of Train_0925 can be found in Materials S1. We constructed a newly determined test set Test_1025 to validate TurnP. Test_1025 was assembled using the same criteria as Train_0925: the PDB of all sequences in 2010, with no two chains sharing more than 25% sequence identity with either the test set or Train_0925. As a result, 736 entries were obtained.

In addition, a database TurnDB_09 was built for users to search real turns of proteins. Sequences in TurnDB_09 were collected from the PDB until December 2009; the sequences had been determined by X-ray crystallography with a resolution better than 2.0 Å. TurnDB_09 contains 48,428 sequences. Turns and secondary structure elements of TurnDB_09 were prepared for users to query.

We identified turns by the coordinate analyzing program Promotif [25] and real secondary structure [26]. Promotif is a program which can locate turns, especially β-turns and γ-turns, from pdb file of a protein. According to the definitions of tight turns [3], most of α- and π-turns were overlapped with γ- or β-turns. In addition, we excluded those α- and π-turns that were in the regular helix regions, which were also excluded by many former researchers [2]. We considered eight-state secondary structure information (obtained with DSSP [26]) as a complement. When there were equal or more than three consecutive “T”, we marked these amino acids as turns. Therefore, we regard our datasets as the collection of all turns of each sequence.

DSSP was used to obtain secondary structure elements. The eight-state elements were designed with the following scheme: H, G, and I to helix; E to sheet; and all others to random coil [27]. The statistical results of the three datasets are listed in Table 1. Moreover, there are 51.22% turns located in the coil region, which is counted based on Train_0925.

**Table 1:** Statistic result of datasets and database.

Dataset	Sequence count	Residue count	Turn count	Turn percentage
**Train_0925**	4,107	929,035	228,062	24.5%
**Test_1025**	736	176,674	42,740	24.2%
**TurnDB_09**	48,428	11,293,117	2,759,778	24.4%
**EVAset1**	77	7,718	1,973	25.6%
**CASP9**	248	53,144	12,336	23.2%

Besides the datasets that generated by our group, two other benchmark datasets were used to test our methods. One is EVAset1 [28], which contains 80 sequences, which are unique and have very low sequence identities against known proteins. This strict criterion makes the dataset a convincing benchmark to evaluate predictors. The other one is CASP9. The biannual CASP experiments present a unique platform for testing new methods through blind predictions. We evaluated our method on the CASP9 data, which was released after May 2010. Each target in the dataset was new to our model, for all sequences which in our training set were released before 2010.

### 2 The Flowchart of TurnP

The flowchart of TurnP is shown in Figure legends.

**Figure 1:**
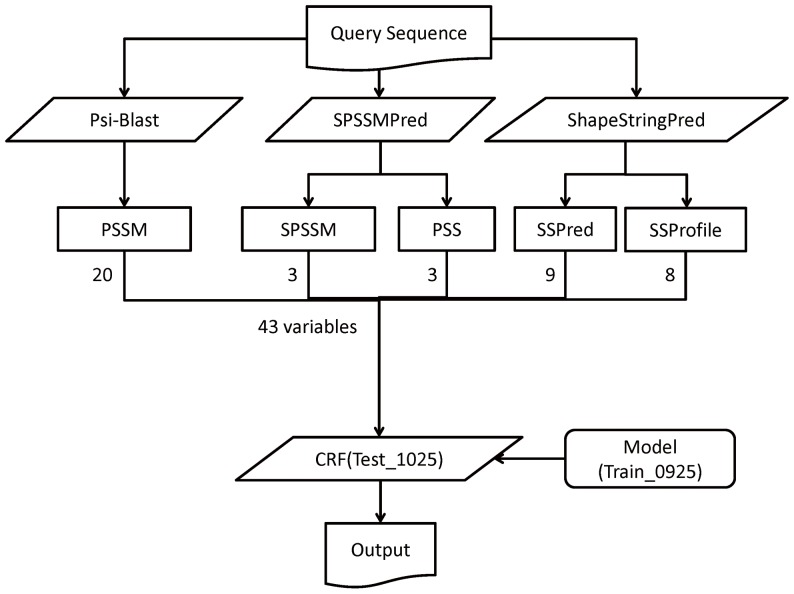
The flowchart of TurnP.

**Figure 1** **.** For a query, the PSI-BLAST, SPSSMPred [20], and a shape string predictor [21] were launched simultaneously. Then, five type features, PSSM, SPSSM, predicted secondary structure (PSS), predicted shape string (SSPred), and shape string profile (SSProfile), were obtained. Finally, all these features, which were combined into a vector of 43 elements for each residue in a sequence, were treated as the input into CRF for modeling and prediction, where the model was trained by Train_0925.

### 3 Protein Secondary Structure Prediction

**Figure 2:**
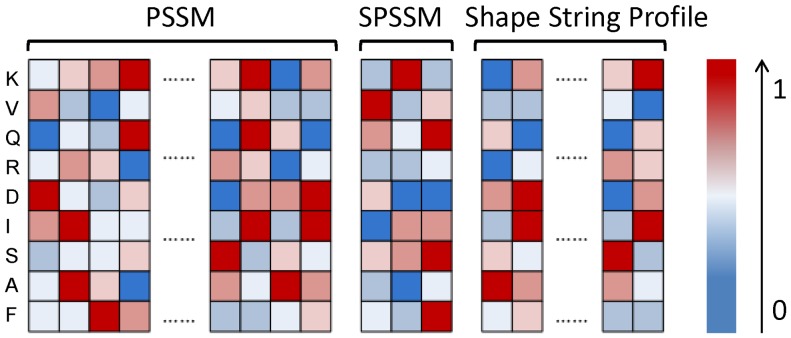
Diagram of profile. We used 3 kinds of profiles in this study: PSSM, SPSSM and Shape string profile. They have 20, 3 and 8 specific elements for each amino acid respectively obtained by sequence alignment and sequence-structure alignment. Each square represents a element that is normalized frequency. The red squares represent large values near ‘1′ and blue ones represent small values near ‘0′; and the deeper the color of the square is, the closer the value to extreme values.

Protein secondary structure information is extremely vital to protein structural prediction. When the structure of a query is unknown, the secondary structure of the sequence could be predicted by many published predictors.

Here, we used a novel predictor, SPSSMPred, which was recently presented by our group [20] to predict the secondary structures of a query. The SPSSMPred was based on an original SPSSM that was generated by sequence alignment, but its elements were secondary structure profiles. The SPSSM can be used to build the relationship between the structural profile and the protein secondary structure. We developed a strategy to construct a database of the secondary structure profiles of 9 million sequences. This database, 9M_database, was one in which every union was an amino acid and its secondary structure profile was derived from the non-redundant database used in PSI-BLAST against a database of known structures. We provided a BLAST tool, 9MBLAST, to simultaneously align a query against the 9M_database and the results in PSSM and SPSSM. A non-redundant dataset was used as the training set. The SPSSMPred was tested on newly published protein sequences and benchmark EVA datasets; we achieved results much closer to the expected theoretical limit of secondary structure prediction (Materials S3). The excellent performance for the prediction of secondary structure is one of the foundations of TurnP.

**Table 2:** 5-fold cross validation results of Train_0925.

	Ac (%)	Q_pred_ (%)	Sn (%)	Sp (%)	MCC
**Without profile features**	87.2	76.7	67.7	93.4	0.64
**All features**	88.8	79.9	71.8	94.2	0.69
**Real variable**	90.3	85.2	72.4	96.0	0.72

**Table 3:** Validation results with Test_1025 as test set.

	Ac (%)	Q_pred_ (%)	Sn (%)	Sp (%)	MCC
**Without profile features**	82.3	64.7	57.6	90.1	0.50
**All features**	82.6	64.4	60.8	89.5	0.51

The first line shows the validation result using PSSM, Predicted Secondary Structure and Predicted Shape String as feature; the second line shows the validation result adding two more structural evolution information: SPSSM and Shape String Profile.

### 4 Protein Shape String Prediction

Backbone dihedral angle is also a characteristic of the secondary structure of a protein, and it is usually described by the φ/Ψ pair in the Ramachandran space and can also be expressed as a shape string [29]. Shape strings contain clustering information, which suggests that they can play an important role in the prediction of protein structures [30].

**Figure 3:**
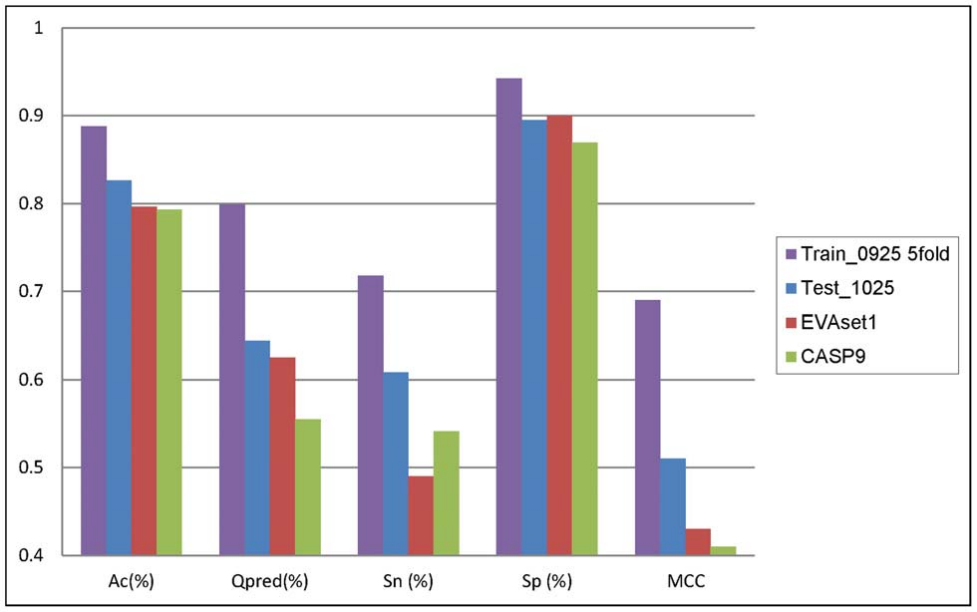
Comparison chart between Train_0925 5-fold validation and evaluation result of Test_1025, EVAset1 and CASP9.

**Table 4:** The prediction performance of our method on EVAset1 and CASP9.

	Ac (%)	Q_pred_ (%)	Sn (%)	Sp (%)	MCC
**EVAset1^a^**	79.6	62.5	49.0	90.0	0.43
**EVAset1^b^**	73.1	48.0	75.7	72.2	0.43
**CASP9^a^**	79.3	55.5	54.1	86.9	0.41
**CASP9^b^**	73.6	45.9	77.2	72.5	0.43

‘a’ represents the original prediction results. ‘b’ represents the prediction results with decision threshold of 0.2.

Here, we used a novel predictor, SSPred, which accurately predicts the shape string of a query as we recently described [21]. The innovative technology is the hallmark pattern library (HPL). The HPL was instrumental in searching structural similarities among highly divergent proteins. A hallmark pattern is composed of short, consecutive sequences that are conserved both in the sequences and in the shape strings. The HPL was believed to reflect remote homologue information in the “twilight zone”. Initially, we began a traversal search for conserved sequence patterns with sufficient frequency in a representative non-redundant PDB chain set **(**NCBI, 2009, 7775 entries**)**. We used an algorithm [31] to extract local combinational variables from unequal-length sequences without sequence alignment. These short patterns were merged with every other single fragment that contained the same residue as the former fragment to form potentially longer sequences while maintaining the frequency criterion. We set the frequency criterion to 100, and 5,667 conserved sequence patterns were obtained. Then, for each position of a conserved sequence pattern, the *p*-value of the corresponding shape string of the amino acid at this position was calculated. If one of the *p*-values of a pattern was less than the background probability, the conserved sequence pattern was identified as a significant hallmark pattern. Finally, based on the *p*-values, we selected 2,761 hallmark patterns that typically exhibited conserved structures to construct the library. The HPL represented remote homology in the sequences and shape strings, and it was an indispensable procedure for our approach to predict shape strings (Materials S4). The improved performance of prediction with the shape string profile is another important element of TurnP.

**Figure 4:**
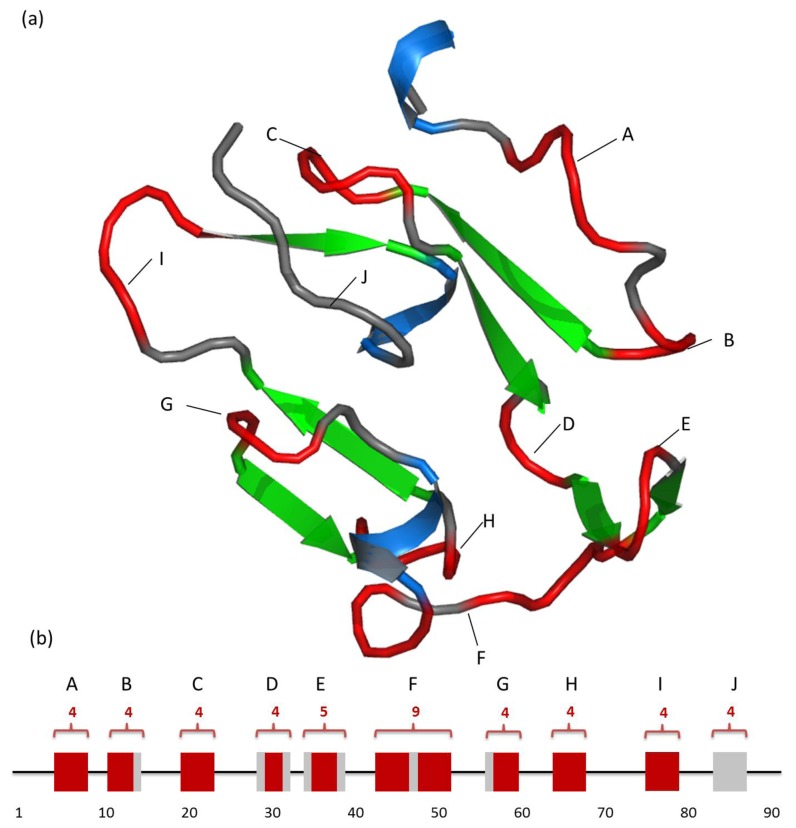
An example of prediction of TurnP, 1B9W_A. in Figure 4(a), the red curve represents turn residues which have been marked as turn, while the other grey curve represents Random Coil. The marine spiral represents Helix, and the green arrow represents Sheet. In the Figure 4(b), all turns in 1B9W_A were shown as blocks to make them more clearly to see. The line represents the sequence, the red blocks represent turn residues which have been predicted correctly, while the grey ones represent turn residues that have not been predicted by TurnP. Position number is counted every 10 residues for convenience, and the position of relevent turns were signed in (a). The illustration of the 3D structure was drawn by PyMOL [34].

**Table 5:** Performance comparison of the present method and other methods.

	Ac (%)	Q_pred_ (%)	Sn (%)	MCC
**TurnP**	88.8	79.9	71.8	0.69
SOM of Meissn**er’s Group**	76.0	81.1	67.8	0.53
**SVM of Meissner’s Group**	61.9	45.0	68.1	0.25

### 5 Profiles

In this work, two new features based on structural evolution information were utilized to improve the accuracy of turn prediction: profiles of secondary structure and shape strings. The profile of the sequences, PSSM, contains rich sequence evolution information; PSSM has proved to be an effective variable for the prediction of protein structure and function. The profiles of secondary structure and shape string are considered to contain structural evolution information and are introduced for the prediction of turn in the protein. These profiles of secondary structure and shape string are generated by sequence-structural alignment. There are three elements of a vector in secondary structure profile and eight elements of a vector in shape string profile for each residue (Figure 2). Initially the sequence of a query is aligned against a special database that contains sequences and known structural information (secondary structures or shape strings). Then top N (say 50) sequences, whose E-values are less than 1E-6, are selected as matched sequences. The structural element of matched sequences is counted at each position of the query. It is the frequency of the structural element at this position after alignment. Finally the normalized vector is output as the structural profile of this residue. The performance of TurnP confirmed that these profiles are useful features for predicting the structure and function of proteins.

### 6 Conditional Random Field

We performed our prediction by applying CRF to predict all turns. CRF is an algorithm for building probabilistic models to segment and label sequence data [22]; CRF is superior to many other machine-learning methods in terms of speed. CRF can exploit context sequence without slide-window when the lengths of sequences are different. It was utilized in our approach for modeling and prediction, with the Unigram template where two upward variables and two downward variables in a row were considered.

### 7 Performance Evaluation

Measures to evaluate the turn/non-turn issue are Ac (also referred to as Q_total_), Q_pred_(also referred to as Q_under_), Sn (also referred to as Q_observed_ or Q_under_), Sp and MCC (Matthew’s Correlation Coefficient), they can be calculated according to following equations:
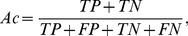
(1)

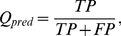
(2)

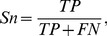
(3)

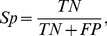
(4)

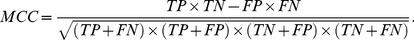
(5)


Here, *TP* is the number of correctly classified turn residues, *TN* is the number of correctly predicted non-turn residues, *FP* is the number of non-turn residues incorrectly classified as turn residues, *FN* is the number of turn residues incorrectly predicted as non-turn residues.

From the definition, it can be found that the Ac is the percentage of correctly predicted residues; the Q_pred_ is the percentage of correctly predicted turns; Sn reflects the percentage of correctly predicted turns among the observed turns; and MCC is a robust measure of the prediction quality, which considers both over- and underpredictions.

## Results and Discussion

### 1 Performances of Cross Validation

The performance of a five-fold cross validation test on Train_0925 is listed in Table 2. We performed validations on the training set using both features with and without the structural evolution information, which was in the shape of SPSSM and shape string profile. With the adding of profile features, the Ac improved from 87.2% to 88.8%. The main contribution of the added variables was improving the Sn, and its increase of 4.1 percentage lead to 0.05 improvement of MCC. These results indicate that the training model using structural evolution information has a strong potential for practical applications. The performance was highly impressive for turn prediction; in addition, the method is uniquely capable of global turn prediction.

As shown in Table 2, an accuracy of 90.3% could be achieved by utilizing the real secondary structures (obtained with DSSP), real shape strings [29], and their profiles as features. The comparision result highlights that the features we chose are very reliable.

### 2 Prediction of Newly Determined Sequences

All other reported methods are used to predicting certain turn categories separately, while our method predicting global turns in proteins. By using optimized model with all features mentioned above, we achieved a better result (Table 3). Even the improvement of accuracy was only 0.3 percentages, the Sn increased from 57.6% to 60.8%, which offset part of the impact of dataset skewedness.

### 3 Evaluation on Benchmark EVA Dataset

To further assess our method, we performed a blind test with the EVAset1; nine sequences that were present in both the EVAset1 and the training model were removed from the Train_0925. Table 4 shows the prediction performance of our method on EVAset1. A Sn of 49.0% was achieved, even for unique protein sequences.

Due to the imbalance between positive and negative data, the specificity is much higher than the sensitivity in Table 4, which occurs frequently in binary classification when the default decision threshold is 0.5. It is possible to adjust the decision threshold to obtain reasonable sensitivity and specificity [32]. Table 4 shows the results that the decision threshold is set as 0.2 for EVAset1 and CASP9 data. It is obviously that the sensitivity is raised and the specificity is debased. However there are two issues that trigger the discussion of adjusting decision threshold: (i) how to optimize decision threshold when the real value is unknown (testing case). It was proposed to determine decision threshold on training sets or datasets whose data was similar with test sets [33]. However, in general the determined decision threshold was not always optimized; (ii) the criterion of adjustment is ambiguous. Table 4 shows that the Ac is worsened after adjustment, and other measurements are affected by adjustment. In fact, adjusting decision threshold is to move a point on a receiver operating characteristics (ROC), it doesn’t change the area under an ROC curve (AUC). Here the results with and without adjustment were both listed for predictions of EVAset1 and CASP9.

### 4 Evaluation on CASP9

As detailed in Table 4, the accuracy of prediction was 79.3%. The result proved that TurnP was among the best methods of Turn Prediction. A comparison chart between Train_0925 5-fold validation and evaluation result of Test_1025, EVAset1 and CASP9 was shown in Figure 3. The chart shows that the overall accuracy hardly fluctuated and all the results were around 80%, which indicates our method is very stable. More detailed results can be found in Material S2.

### 5 Prediction of the Turns in Merozoite Surface Protein

1B9W_A, which is the crystal structure of the C-terminal of the merozoite surface protein, was used as an example to test one of the predicted results of TurnP. 1B9W_A is essential for successful erythrocyte invasion by the malaria parasite, *Plasmodium*. There are 91 residues in total, which form ten turns, and nine turns were hinted. The lengths of these turns are from 4−9 residues; all the turns are marked in Figure 4.

### 6 Performance Comparison with Methods of Turn Category Prediction

Table 5 shows the performance comparison between TurnP and a prediction method of Meissner’s group [2], which using self-organizing map (SOM) and SVM with uniform classification to predict all turn families. The MCC of TurnP valued up to 0.69, which shown the excellent ability of turn prediction.

### 7 Web Server

We have set up a web server on our local infrastructure for the use of TurnP by scientific researchers. It is available at http://cal.tongji.edu.cn/TurnP/index.jsp. There are two parts in TurnP: analysis and prediction.

In the analysis section, users are allowed to search and display the turns of proteins among the TurnDB_09 online via submitting a PDB ID or download the entire database in a text format. The output of searching as well as a statistical analysis of the amount of consecutive turn residues in different lengths can be seen in the Material S5.

Prediction section provides a powerful predictor of all types of turns for a query or a file (FASTA format). An ID is required to mark each entry, and a random ID can be given when a user submits only one query without an ID. The output of a query sequence consists of predicted turns and the probability of the turn prediction. The results of the turn and non-turn are identified by different colors for easy discrimination. To visualize the results, we created a concise and vivid colorful string as the secondary structure diagram, which can be displayed with low software requirements.

### Conclusions

In this study, we proposed the approach for predicting all the types of turns in a protein. The turns of a protein compose a tight structure based on common mechanisms; thus, turns and non-turns can be identified based on a unified model. The innovation technology was the utilization of structural evolution information. We introduced two novel features: SPSSM and shape string profiles, which can accurately reflect the characteristics of turns. Hence, these novel features were beneficial to the performance of the predictor. We constructed a prediction model by a non-redundant dataset and tested the model on a newly determined dataset, the benchmark EVA set as well as CASP9 data. The results showed that TurnP outperformed existing models in the field of large-scale turn prediction. TurnP is a pioneering work for the prediction of global turns throughout a protein; we believe it can be complementary to other protein secondary structure prediction methods and may be useful for protein three-dimensional structure prediction.

## Supporting Information

Figure S1 **An example of generation of raw SPSSM in 9M_database. A query in 9M_database is sp|P85173.1, and its listed 49 amino acids (first line). Four aligned ‘Sbjcts’ examples (in PDB_99) are shown, and two arrow tips point to two obtained sequences and their secondary structures. Then the query and found secondary structural elements are shown in middle. After score, its raw SPSSM is constructed and a part of them (in red) is shown in bottom.**
(TIF)

Figure S2 **Schemes of SSPred. (A) The flowchart of the prediction of shape string and (B) sequence alignment with hallmark patterns as seeds. An example of (C) the predicted shape string and (D) the output sequence shape string profile. AA: amino acid; MT, match times; PredSS, predicted shape string; Prob, output probability.**
(TIF)

Figure S3 1AOP_A was taken as an example of analysis result. The output contains sequence, turn, secondary structure as well as a colorful string diagram of sequence’s main secondary structures ('-'for band, '+'for turn, ' = 'for beta-sheet and '*'for alfa-helix).
(TIF)

Figure S4 An example of TurnP output. Each output of a query is corresponding to 9 lines: sequence, position number; predicted turn, predicted three-state secondary structure elements, predicted shape string and probability of each prediction; structure diagram string. In the diagram, characters are schemed as follow: ‘-‘ for band, ‘+’ for turn, ‘ = ’ for β-sheet and ‘*’ for α-helix.
(TIF)

Table S1 **PDB ID list of Train_0925.**
(DOCX)

Table S2 **Detailed results of datasets.**
(DOCX)

Text S1 **PDB ID list of Train_0925.**
(DOCX)

Text S2 **Details of results.**
(DOCX)

Text S3 **Secondary structure prediction-SPSSMPred.**
(DOCX)

Text S4 **Shape string prediction-SSPred.**
(DOCX)

Text S5 **Webserver introduction.**
(DOCX)
