# Supplementary figures and images for: Predicting Turns in Proteins with a Unified Model
Source: PLoS One. 2012 Nov 7;7(11):e48389. doi: 10.1371/journal.pone.0048389 (PMC3492357; doi:10.1371/journal.pone.0048389)

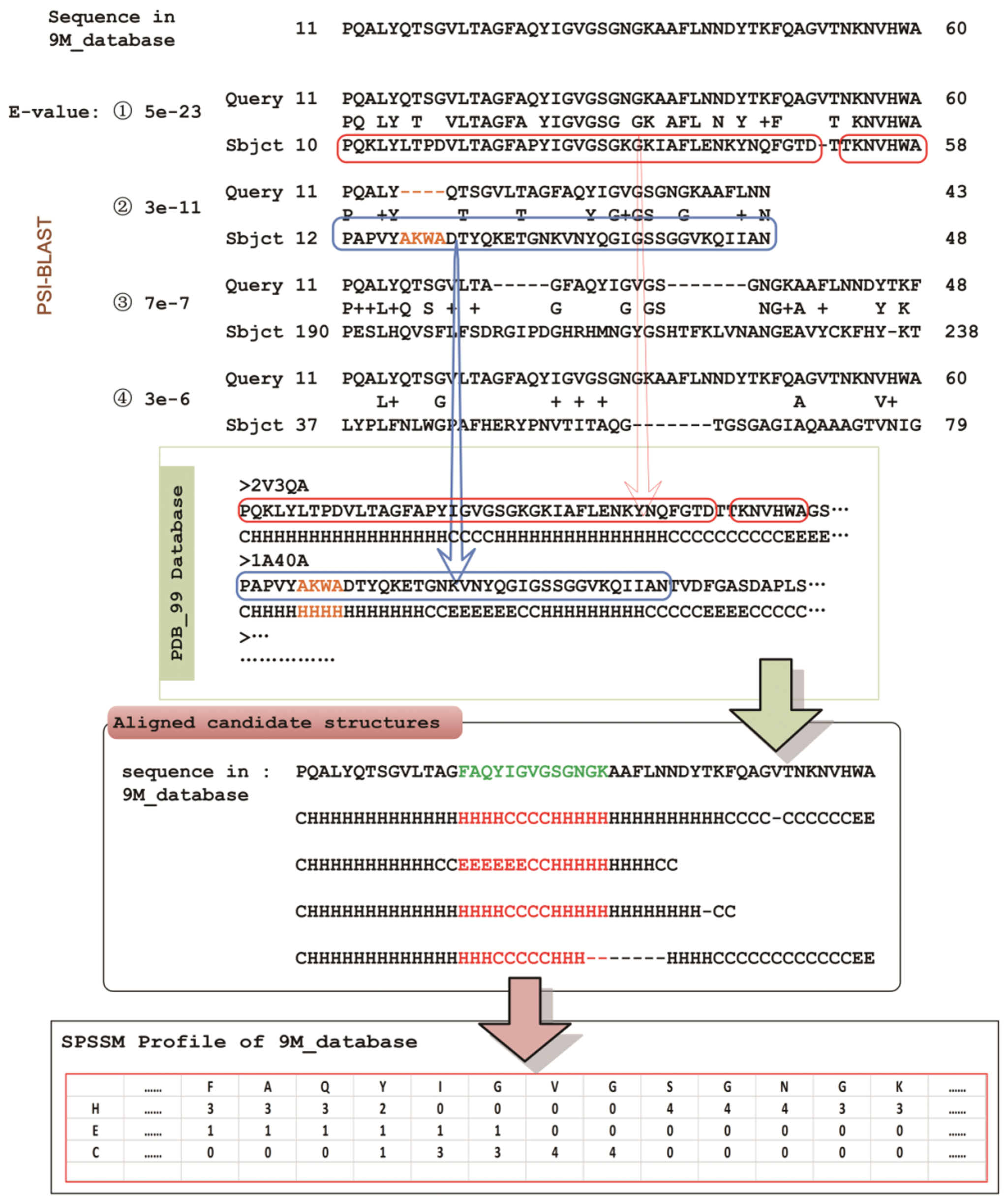

Supplement: Figure S1 — An example of generation of raw SPSSM in 9M_database. A query in 9M_database is sp|P85173.1, and its listed 49 amino acids (first line). Four aligned ‘Sbjcts’ examples (in PDB_99) are shown, and two arrow tips point to two obtained sequences and their secondary structures. Then the query and found secondary structural elements are shown in middle. After score, its raw SPSSM is constructed and a part of them (in red) is shown in bottom. (TIF) [file pone.0048389.s001.tif]

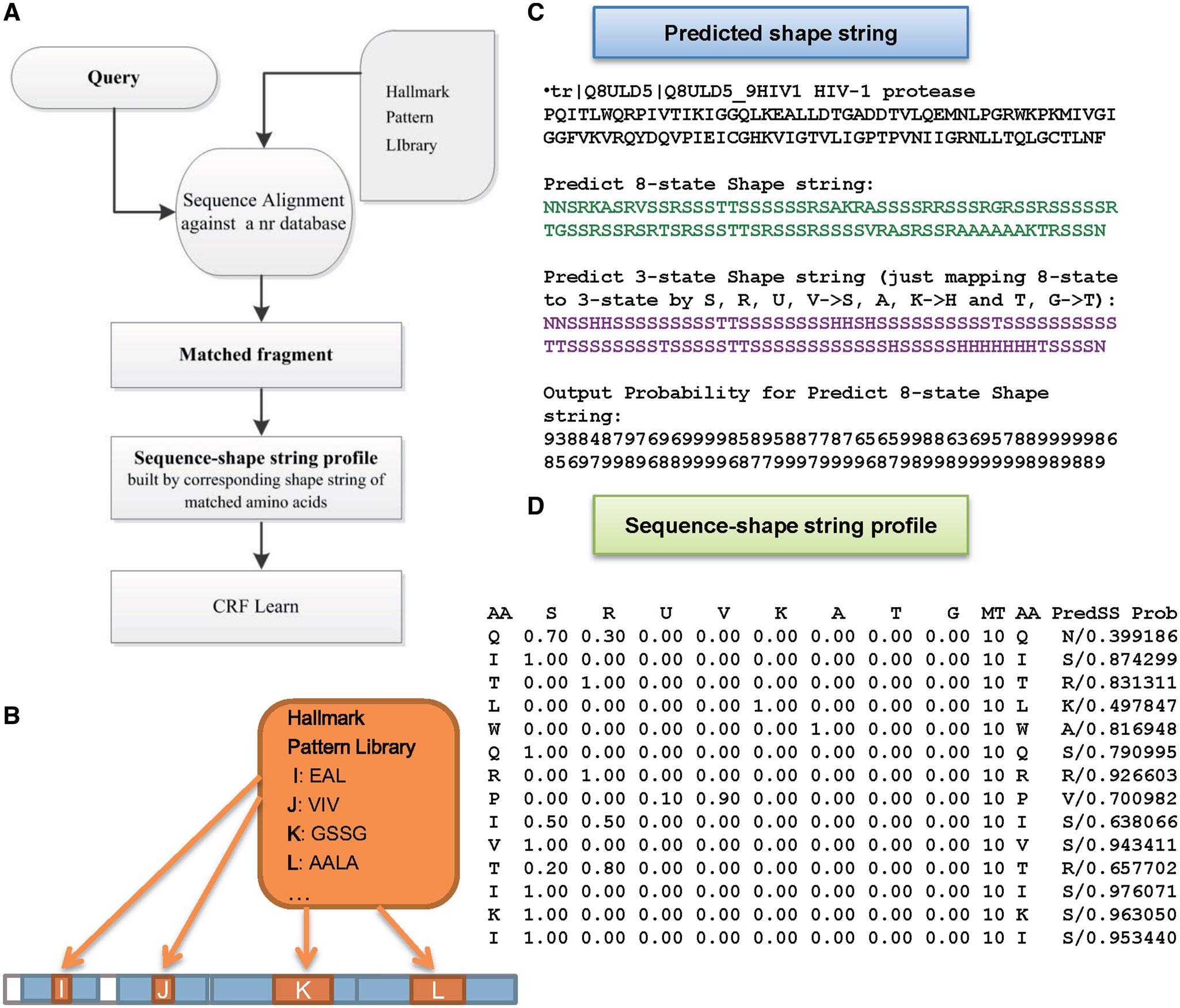

Supplement: Figure S2 — Schemes of SSPred. (A) The flowchart of the prediction of shape string and (B) sequence alignment with hallmark patterns as seeds. An example of (C) the predicted shape string and (D) the output sequence shape string profile. AA: amino acid; MT, match times; PredSS, predicted shape string; Prob, output probability. (TIF) [file pone.0048389.s002.tif]

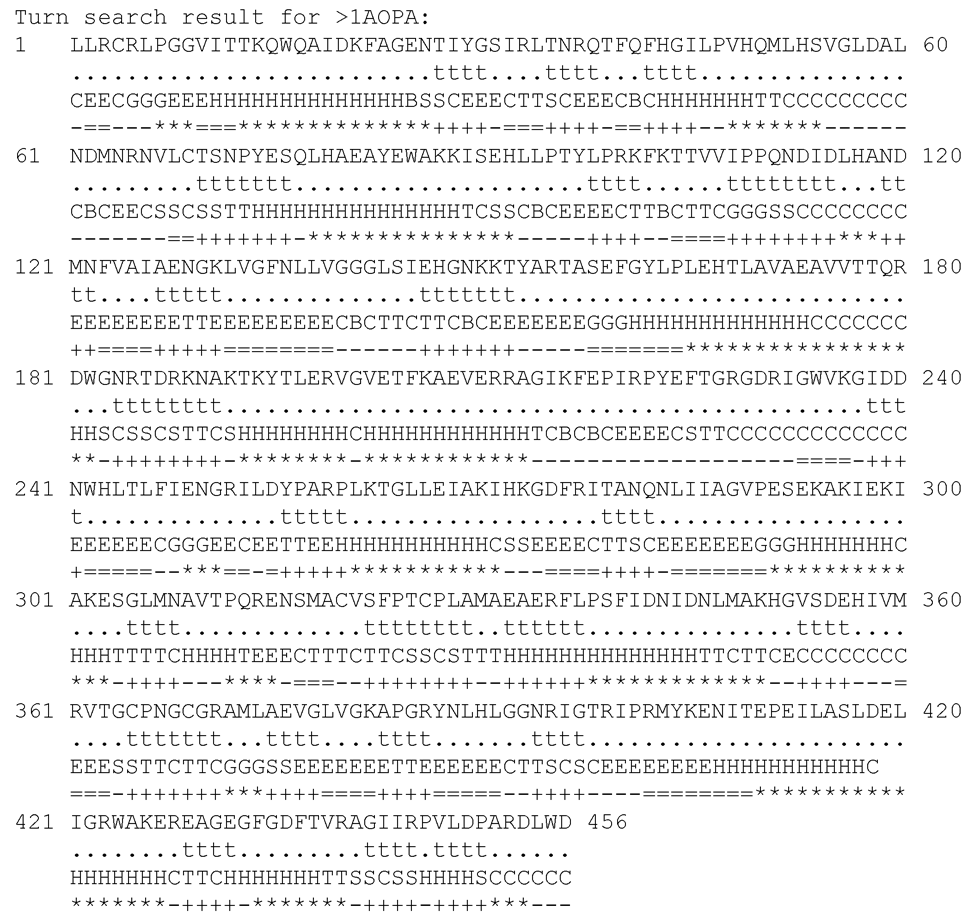

Supplement: Figure S3 — 1AOP_A was taken as an example of analysis result. The output contains sequence, turn, secondary structure as well as a colorful string diagram of sequence’s main secondary structures ('-'for band, '+'for turn, ' = 'for beta-sheet and '*'for alfa-helix). (TIF) [file pone.0048389.s003.tif]

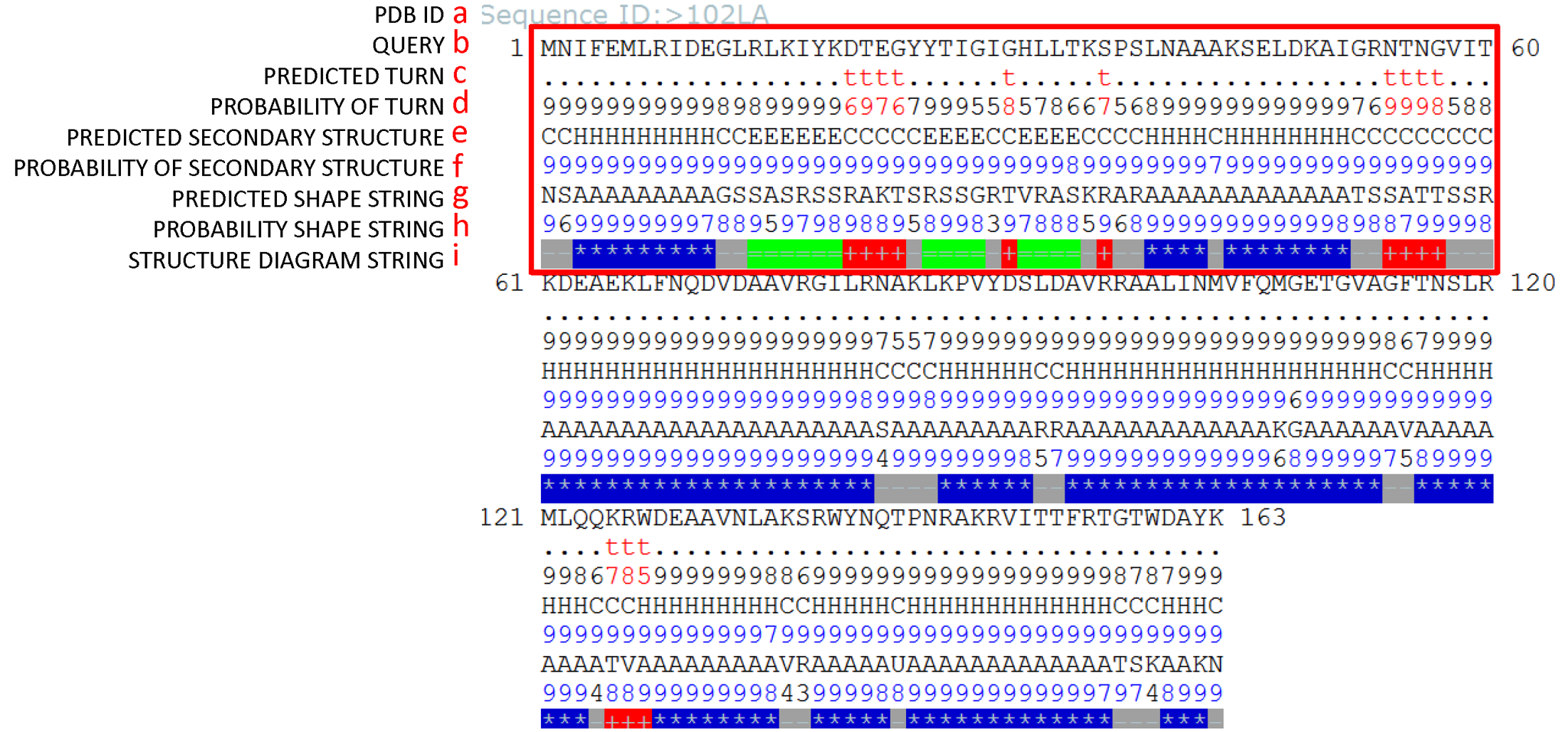

Supplement: Figure S4 — An example of TurnP output. Each output of a query is corresponding to 9 lines: sequence, position number; predicted turn, predicted three-state secondary structure elements, predicted shape string and probability of each prediction; structure diagram string. In the diagram, characters are schemed as follow: ‘-‘ for band, ‘+’ for turn, ‘ = ’ for β-sheet and ‘*’ for α-helix. (TIF) [file pone.0048389.s004.tif]
