# Supplementary material for: Predicting Turns in Proteins with a Unified Model
Source: PLoS One. 2012 Nov 7;7(11):e48389. doi: 10.1371/journal.pone.0048389 (PMC3492357; doi:10.1371/journal.pone.0048389)
Supplement: Table S1 — PDB ID list of Train_0925. (DOCX) [file pone.0048389.s005.docx]

Table S1. PDB ID list of Train_0925

| PDB ID | PDB ID | PDB ID | PDB ID | PDB ID | PDB ID | PDB ID |
| --- | --- | --- | --- | --- | --- | --- |
| \| 1A12A \| \| --- \| \| 1A1XA \| \| 1A3AA \| \| 1A3CA \| \| 1A4IA \| \| 1A53A \| \| 1A62A \| \| 1A8DA \| \| 1A8LA \| \| 1A92A \| \| 1A9XB \| \| 1ABAA \| \| 1AE9A \| \| 1AH7A \| \| 1AHOA \| \| 1AK0A \| \| 1AL3A \| \| 1ALUA \| \| 1AMFA \| \| 1AOCA \| \| 1AOLA \| \| 1AOPA \| \| 1AQZA \| \| 1ARBA \| \| 1ATGA \| \| 1ATZA \| \| 1AYOA \| \| 1AZOA \| \| 1B0NB \| \| 1B12A \| \| 1B1CA \| \| 1B25A \| \| 1B3AA \| \| 1B43A \| \| 1B4FA \| \| 1B5EA \| \| 1B6AA \| \| 1B8ZA \| | \| 1B9WA \| \| --- \| \| 1BAMA \| \| 1BAZA \| \| 1BEAA \| \| 1BF2A \| \| 1BGCA \| \| 1BGFA \| \| 1BKRA \| \| 1BM8A \| \| 1BRTA \| \| 1BTKA \| \| 1BTNA \| \| 1BU8A \| \| 1BUPA \| \| 1BX7A \| \| 1BXYA \| \| 1BYIA \| \| 1BYRA \| \| 1C1DA \| \| 1C1KA \| \| 1C1LA \| \| 1C1YB \| \| 1C3CA \| \| 1C4QA \| \| 1C5EA \| \| 1C75A \| \| 1C7KA \| \| 1C7SA \| \| 1C96A \| \| 1CB8A \| \| 1CC8A \| \| 1CCWA \| \| 1CCWB \| \| 1CDCA \| \| 1CEOA \| \| 1CEWI \| \| 1CFBA \| \| 1CHDA \| | \| 1CHMA \| \| --- \| \| 1CI4A \| \| 1CMCA \| \| 1COZA \| \| 1CQ3A \| \| 1CQMA \| \| 1CQQA \| \| 1CQYA \| \| 1CS1A \| \| 1CTFA \| \| 1CUJA \| \| 1CV8A \| \| 1CVRA \| \| 1CXQA \| \| 1CY5A \| \| 1CZPA \| \| 1CZYA \| \| 1D0CA \| \| 1D0DA \| \| 1D0QA \| \| 1D2NA \| \| 1D2OA \| \| 1D2SA \| \| 1D2TA \| \| 1D2VA \| \| 1D2VC \| \| 1D3BB \| \| 1D3YA \| \| 1D4OA \| \| 1D4TA \| \| 1D5TA \| \| 1D7PM \| \| 1D8HA \| \| 1D8WA \| \| 1DCIA \| \| 1DCSA \| \| 1DD9A \| \| 1DDWA \| | \| 1DEKA \| \| --- \| \| 1DF4A \| \| 1DG6A \| \| 1DGWX \| \| 1DGWY \| \| 1DJ0A \| \| 1DJ8A \| \| 1DK8A \| \| 1DLWA \| \| 1DMGA \| \| 1DMHA \| \| 1DNLA \| \| 1DOWA \| \| 1DP4A \| \| 1DPGA \| \| 1DQGA \| \| 1DQPA \| \| 1DQTA \| \| 1DQZA \| \| 1DS1A \| \| 1DTDB \| \| 1DUVG \| \| 1DVOA \| \| 1DWKA \| \| 1DY5A \| \| 1DZFA \| \| 1DZKA \| \| 1E19A \| \| 1E29A \| \| 1E2KA \| \| 1E2WA \| \| 1E4CP \| \| 1E4FT \| \| 1E58A \| \| 1E5KA \| \| 1E6UA \| \| 1E7LA \| \| 1E8CA \| | \| 1E8YA \| \| --- \| \| 1EAQA \| \| 1EARA \| \| 1EAYC \| \| 1EB6A \| \| 1ECAA \| \| 1EE8A \| \| 1EERA \| \| 1EEXA \| \| 1EEXB \| \| 1EEXG \| \| 1EF1C \| \| 1EF8A \| \| 1EFDN \| \| 1EJDA \| \| 1EJGA \| \| 1EKQA \| \| 1EL6A \| \| 1ELKA \| \| 1ELUA \| \| 1ELWA \| \| 1EOKA \| \| 1EQ2A \| \| 1ERZA \| \| 1ES5A \| \| 1ES6A \| \| 1ES9A \| \| 1EU1A \| \| 1EU8A \| \| 1EUVA \| \| 1EUWA \| \| 1EVFA \| \| 1EVLA \| \| 1EVXA \| \| 1EW4A \| \| 1EWFA \| \| 1EX0A \| \| 1EX2A \| | \| 1EXTA \| \| --- \| \| 1EYBA \| \| 1EYHA \| \| 1EYQA \| \| 1EZ3A \| \| 1EZGA \| \| 1EZIA \| \| 1EZWA \| \| 1F00I \| \| 1F0XA \| \| 1F1EA \| \| 1F1MA \| \| 1F2DA \| \| 1F2LA \| \| 1F2TB \| \| 1F32A \| \| 1F3UA \| \| 1F3UB \| \| 1F3VA \| \| 1F46A \| \| 1F5MA \| \| 1F5NA \| \| 1F5VA \| \| 1F60B \| \| 1F7LA \| \| 1F86A \| \| 1F8EA \| \| 1F94A \| \| 1F9VA \| \| 1FC3A \| \| 1FCQA \| \| 1FD0A \| \| 1FD3A \| \| 1FIPA \| \| 1FITA \| \| 1FJ2A \| \| 1FK5A \| \| 1FKMA \| | \| 1FL0A \| \| --- \| \| 1FLMA \| \| 1FM0D \| \| 1FM0E \| \| 1FN9A \| \| 1FNNA \| \| 1FO8A \| \| 1FOBA \| \| 1FP2A \| \| 1FPOA \| \| 1FS7A \| \| 1FSGA \| \| 1FT5A \| \| 1FTRA \| \| 1FVIA \| \| 1FX2A \| \| 1FXOA \| \| 1FYEA \| \| 1G12A \| \| 1G1TA \| \| 1G2RA \| \| 1G3KA \| \| 1G3PA \| \| 1G4MA \| \| 1G4YB \| \| 1G55A \| \| 1G5AA \| \| 1G5HA \| \| 1G60A \| \| 1G61A \| \| 1G66A \| \| 1G6HA \| \| 1G6UA \| \| 1G6XA \| \| 1G73A \| \| 1G7SA \| \| 1G8EA \| \| 1G8KA \| |
| \| 1G8LA \| \| --- \| \| 1G8MA \| \| 1G8QA \| \| 1G9GA \| \| 1GA6A \| \| 1GA8A \| \| 1GAKA \| \| 1GBSA \| \| 1GCIA \| \| 1GK9A \| \| 1GK9B \| \| 1GKMA \| \| 1GKPA \| \| 1GL2C \| \| 1GMUA \| \| 1GMXA \| \| 1GNLA \| \| 1GNYA \| \| 1GO3F \| \| 1GOTG \| \| 1GP0A \| \| 1GP6A \| \| 1GPPA \| \| 1GPRA \| \| 1GQEA \| \| 1GQIA \| \| 1GS5A \| \| 1GS9A \| \| 1GSAA \| \| 1GSOA \| \| 1GTKA \| \| 1GTTA \| \| 1GTVA \| \| 1GU2A \| \| 1GU7A \| \| 1GUDA \| \| 1GUQA \| \| 1GUTA \| \| 1GV9A \| \| 1GVDA \| \| 1GVJA \| \| 1GVNA \| \| 1GVPA \| \| 1GWEA \| | \| 1GWMA \| \| --- \| \| 1GWUA \| \| 1GWYA \| \| 1GXJA \| \| 1GXMA \| \| 1GXRA \| \| 1GXUA \| \| 1GXYA \| \| 1GY7A \| \| 1GYXA \| \| 1H03P \| \| 1H05A \| \| 1H0HB \| \| 1H16A \| \| 1H1NA \| \| 1H2SB \| \| 1H2VC \| \| 1H2WA \| \| 1H32A \| \| 1H4AX \| \| 1H4GA \| \| 1H4RA \| \| 1H4XA \| \| 1H6LA \| \| 1H72C \| \| 1H7CA \| \| 1H7EA \| \| 1H8PA \| \| 1H97A \| \| 1H99A \| \| 1HBNA \| \| 1HBNB \| \| 1HBNC \| \| 1HDHA \| \| 1HDOA \| \| 1HE1A \| \| 1HF8A \| \| 1HFES \| \| 1HH8A \| \| 1HHSA \| \| 1HM9A \| \| 1HN0A \| \| 1HNJA \| \| 1HP1A \| | \| 1HQ0A \| \| --- \| \| 1HQSA \| \| 1HTRP \| \| 1HUFA \| \| 1HUWA \| \| 1HX0A \| \| 1HX1B \| \| 1HX6A \| \| 1HXIA \| \| 1HXNA \| \| 1HXRA \| \| 1HYOA \| \| 1HYPA \| \| 1HZ4A \| \| 1HZ6A \| \| 1HZTA \| \| 1I0RA \| \| 1I0VA \| \| 1I12A \| \| 1I1JA \| \| 1I1WA \| \| 1I24A \| \| 1I27A \| \| 1I2AA \| \| 1I2KA \| \| 1I2TA \| \| 1I36A \| \| 1I3CA \| \| 1I4JA \| \| 1I4MA \| \| 1I4UA \| \| 1I52A \| \| 1I58A \| \| 1I60A \| \| 1I6LA \| \| 1I71A \| \| 1I7WB \| \| 1I88A \| \| 1I8AA \| \| 1I8OA \| \| 1IA9A \| \| 1IAPA \| \| 1IBYA \| \| 1IC2A \| | \| 1IDPA \| \| --- \| \| 1IFRA \| \| 1IG0A \| \| 1IGQA \| \| 1IHZA \| \| 1II5A \| \| 1IIBA \| \| 1IJBA \| \| 1IJYA \| \| 1IM5A \| \| 1IN4A \| \| 1INLA \| \| 1IO0A \| \| 1IOMA \| \| 1IQZA \| \| 1IRQA \| \| 1ISPA \| \| 1ISUA \| \| 1ITVA \| \| 1ITXA \| \| 1IUQA \| \| 1IWLA \| \| 1IWMA \| \| 1IX9A \| \| 1IZCA \| \| 1IZMA \| \| 1J0HA \| \| 1J0PA \| \| 1J1NA \| \| 1J1TA \| \| 1J24A \| \| 1J27A \| \| 1J2JB \| \| 1J2LA \| \| 1J2RA \| \| 1J30A \| \| 1J34C \| \| 1J3AA \| \| 1J3WA \| \| 1J5PA \| \| 1J5UA \| \| 1J5WA \| \| 1J77A \| \| 1J7XA \| | \| 1J8BA \| \| --- \| \| 1J8RA \| \| 1J98A \| \| 1JBEA \| \| 1JBOA \| \| 1JCDA \| \| 1JDHA \| \| 1JDWA \| \| 1JE5A \| \| 1JEKA \| \| 1JETA \| \| 1JF3A \| \| 1JF8A \| \| 1JFBA \| \| 1JG1A \| \| 1JH6A \| \| 1JHFA \| \| 1JHGA \| \| 1JHJA \| \| 1JHSA \| \| 1JI7A \| \| 1JIWI \| \| 1JIXA \| \| 1JKEA \| \| 1JKXA \| \| 1JL0A \| \| 1JL1A \| \| 1JM0A \| \| 1JMKC \| \| 1JMVA \| \| 1JMXB \| \| 1JNDA \| \| 1JNIA \| \| 1JO0A \| \| 1JOSA \| \| 1JOVA \| \| 1JQ5A \| \| 1JR2A \| \| 1JR7A \| \| 1JR8A \| \| 1JSDA \| \| 1JSDB \| \| 1JU3A \| \| 1JUVA \| | \| 1JWQA \| \| --- \| \| 1JX6A \| \| 1JY1A \| \| 1JY2N \| \| 1JY2O \| \| 1JY2P \| \| 1JYAA \| \| 1JYEA \| \| 1JYHA \| \| 1JYKA \| \| 1JYOA \| \| 1JYOE \| \| 1JZTA \| \| 1K04A \| \| 1K07A \| \| 1K0MA \| \| 1K2XA \| \| 1K2XB \| \| 1K32A \| \| 1K3SA \| \| 1K3YA \| \| 1K4IA \| \| 1K4NA \| \| 1K55C \| \| 1K5CA \| \| 1K5NA \| \| 1K77A \| \| 1K7CA \| \| 1K7JA \| \| 1K7WA \| \| 1K8KC \| \| 1K8KD \| \| 1K8KE \| \| 1K8KF \| \| 1K8KG \| \| 1K92A \| \| 1KA1A \| \| 1KAEA \| \| 1KAFA \| \| 1KCMA \| \| 1KGDA \| \| 1KHCA \| \| 1KHXA \| \| 1KHYA \| | \| 1KIDA \| \| --- \| \| 1KJQA \| \| 1KKOA \| \| 1KLXA \| \| 1KMOA \| \| 1KMTA \| \| 1KMVA \| \| 1KNGA \| \| 1KOEA \| \| 1KOLA \| \| 1KP6A \| \| 1KPFA \| \| 1KPTA \| \| 1KQ1A \| \| 1KQ6A \| \| 1KQFC \| \| 1KQPA \| \| 1KQRA \| \| 1KS8A \| \| 1KS9A \| \| 1KSHB \| \| 1KT6A \| \| 1KU3A \| \| 1KV7A \| \| 1KVEA \| \| 1KVEB \| \| 1KW4A \| \| 1KWFA \| \| 1KXOA \| \| 1KYFA \| \| 1KZFA \| \| 1KZQA \| \| 1L1EA \| \| 1L1LA \| \| 1L3KA \| \| 1L3PA \| \| 1L5OA \| \| 1L6PA \| \| 1L6RA \| \| 1L7AA \| \| 1L7DA \| \| 1L7LA \| \| 1L9LA \| \| 1L9XA \| |
| \| 1LAMA \| \| --- \| \| 1LBUA \| \| 1LC0A \| \| 1LC5A \| \| 1LDDA \| \| 1LFPA \| \| 1LFWA \| \| 1LKIA \| \| 1LKKA \| \| 1LL2A \| \| 1LLFA \| \| 1LM5A \| \| 1LM8B \| \| 1LM8V \| \| 1LMIA \| \| 1LMLA \| \| 1LNIA \| \| 1LO7A \| \| 1LQTA \| \| 1LQVA \| \| 1LS1A \| \| 1LSHA \| \| 1LSHB \| \| 1LSLA \| \| 1LSTA \| \| 1LTZA \| \| 1LU4A \| \| 1LUCA \| \| 1LUGA \| \| 1LUZA \| \| 1LV7A \| \| 1LWBA \| \| 1LXJA \| \| 1LYQA \| \| 1LYVA \| \| 1LZLA \| \| 1M0DA \| \| 1M0KA \| \| 1M0WA \| \| 1M15A \| \| 1M1FA \| \| 1M1HA \| \| 1M1NA \| \| 1M1NB \| | \| 1M1QA \| \| --- \| \| 1M22A \| \| 1M2DA \| \| 1M40A \| \| 1M48A \| \| 1M4JA \| \| 1M4LA \| \| 1M4TA \| \| 1M55A \| \| 1M5WA \| \| 1M65A \| \| 1M7YA \| \| 1M93C \| \| 1M9ZA \| \| 1MAIA \| \| 1MBMA \| \| 1MC2A \| \| 1MDCA \| \| 1MF7A \| \| 1MG4A \| \| 1MG7A \| \| 1MGTA \| \| 1MIXA \| \| 1MJ4A \| \| 1MJ5A \| \| 1MK0A \| \| 1MK4A \| \| 1MKAA \| \| 1MKKA \| \| 1MLAA \| \| 1MMLA \| \| 1MN8A \| \| 1MOFA \| \| 1MPGA \| \| 1MQOA \| \| 1MSCA \| \| 1MTPA \| \| 1MTPB \| \| 1MTYB \| \| 1MTYD \| \| 1MTYG \| \| 1MUNA \| \| 1MUWA \| \| 1MV8A \| | \| 1MVFD \| \| --- \| \| 1MVLA \| \| 1MW9X \| \| 1MWPA \| \| 1MWQA \| \| 1MXIA \| \| 1MXRA \| \| 1MY7A \| \| 1MZ9A \| \| 1N08A \| \| 1N0QA \| \| 1N0WA \| \| 1N13A \| \| 1N13B \| \| 1N1FA \| \| 1N1JA \| \| 1N2DC \| \| 1N2ZA \| \| 1N3LA \| \| 1N40A \| \| 1N4WA \| \| 1N5UA \| \| 1N62A \| \| 1N62B \| \| 1N62C \| \| 1N7SA \| \| 1N7SB \| \| 1N7SC \| \| 1N7SD \| \| 1N7ZA \| \| 1N8VA \| \| 1N93X \| \| 1N9PA \| \| 1NBAA \| \| 1NC5A \| \| 1NE9A \| \| 1NEPA \| \| 1NF9A \| \| 1NFPA \| \| 1NG6A \| \| 1NIGA \| \| 1NIJA \| \| 1NJHA \| \| 1NJRA \| | \| 1NKDA \| \| --- \| \| 1NKGA \| \| 1NKIA \| \| 1NKRA \| \| 1NKZA \| \| 1NKZB \| \| 1NLNA \| \| 1NLQA \| \| 1NLSA \| \| 1NNFA \| \| 1NNLA \| \| 1NNWA \| \| 1NNXA \| \| 1NOFA \| \| 1NP6A \| \| 1NPYA \| \| 1NQJA \| \| 1NR0A \| \| 1NRJA \| \| 1NRJB \| \| 1NSJA \| \| 1NSZA \| \| 1NTHA \| \| 1NTVA \| \| 1NTYA \| \| 1NU0A \| \| 1NU4A \| \| 1NUYA \| \| 1NWAA \| \| 1NWWA \| \| 1NWZA \| \| 1NXMA \| \| 1NYCA \| \| 1NYKA \| \| 1NYTA \| \| 1NZ0A \| \| 1NZJA \| \| 1NZYA \| \| 1O08A \| \| 1O13A \| \| 1O1YA \| \| 1O22A \| \| 1O26A \| \| 1O2DA \| | \| 1O4TA \| \| --- \| \| 1O4WA \| \| 1O66A \| \| 1O6AA \| \| 1O6DA \| \| 1O75A \| \| 1O7IA \| \| 1O7JA \| \| 1O7QA \| \| 1O82A \| \| 1O8BA \| \| 1O97C \| \| 1O97D \| \| 1OA8A \| \| 1OAIA \| \| 1OAOC \| \| 1OBBA \| \| 1OBOA \| \| 1OCYA \| \| 1OD3A \| \| 1OD6A \| \| 1ODMA \| \| 1OEWA \| \| 1OEYA \| \| 1OEYJ \| \| 1OF8A \| \| 1OFCX \| \| 1OFLA \| \| 1OGDA \| \| 1OGOX \| \| 1OGQA \| \| 1OH0A \| \| 1OI0A \| \| 1OI2A \| \| 1OI7A \| \| 1OIHA \| \| 1OISA \| \| 1OJHA \| \| 1OK0A \| \| 1OK7A \| \| 1OKSA \| \| 1OLZA \| \| 1ON3A \| \| 1ONWA \| | \| 1OO0A \| \| --- \| \| 1OO0B \| \| 1OOEA \| \| 1OOHA \| \| 1OOYA \| \| 1OPCA \| \| 1OQ1A \| \| 1OQJA \| \| 1OQVA \| \| 1OR7C \| \| 1ORSC \| \| 1ORUA \| \| 1OSYA \| \| 1OTKA \| \| 1OU8A \| \| 1OW1A \| \| 1OW4A \| \| 1OWLA \| \| 1OX0A \| \| 1OYGA \| \| 1OYWA \| \| 1OZ2A \| \| 1P1JA \| \| 1P1MA \| \| 1P1XA \| \| 1P3CA \| \| 1P3DA \| \| 1P57A \| \| 1P5DX \| \| 1P5VA \| \| 1P5VB \| \| 1P5ZB \| \| 1P6OA \| \| 1P7TA \| \| 1P90A \| \| 1P9GA \| \| 1P9HA \| \| 1PBJA \| \| 1PBWA \| \| 1PBYA \| \| 1PBYC \| \| 1PCFA \| \| 1PDOA \| \| 1PE9A \| | \| 1PFBA \| \| --- \| \| 1PG4A \| \| 1PG6A \| \| 1PGSA \| \| 1PINA \| \| 1PJXA \| \| 1PK6A \| \| 1PKHA \| \| 1PL3A \| \| 1PM4A \| \| 1PMHX \| \| 1PO5A \| \| 1POCA \| \| 1PP0A \| \| 1PQ7A \| \| 1PQHA \| \| 1PSRA \| \| 1PSWA \| \| 1PTMA \| \| 1PU6A \| \| 1PUCA \| \| 1PV5A \| \| 1PVGA \| \| 1PX4A \| \| 1PX5A \| \| 1PZ4A \| \| 1PZ7A \| \| 1PZWA \| \| 1Q0PA \| \| 1Q0RA \| \| 1Q16A \| \| 1Q1FA \| \| 1Q2HA \| \| 1Q33A \| \| 1Q35A \| \| 1Q40B \| \| 1Q5YA \| \| 1Q5ZA \| \| 1Q6OA \| \| 1Q6ZA \| \| 1Q7EA \| \| 1Q7FA \| \| 1Q7LA \| \| 1Q7LB \| |
| \| 1Q8BA \| \| --- \| \| 1Q8DA \| \| 1Q8FA \| \| 1Q8IA \| \| 1Q9UA \| \| 1QAZA \| \| 1QB5D \| \| 1QCSA \| \| 1QD1A \| \| 1QDDA \| \| 1QF8A \| \| 1QG8A \| \| 1QGEE \| \| 1QGIA \| \| 1QGVA \| \| 1QHDA \| \| 1QJ8A \| \| 1QJPA \| \| 1QKRA \| \| 1QKSA \| \| 1QL0A \| \| 1QLMA \| \| 1QLWA \| \| 1QMYA \| \| 1QNRA \| \| 1QO2A \| \| 1QOPB \| \| 1QOYA \| \| 1QQ5A \| \| 1QQFA \| \| 1QQP1 \| \| 1QQP2 \| \| 1QQP3 \| \| 1QQP4 \| \| 1QR0A \| \| 1QREA \| \| 1QSTA \| \| 1QTWA \| \| 1QU9A \| \| 1QUSA \| \| 1QV9A \| \| 1QW2A \| \| 1QW9A \| \| 1QWGA \| | \| 1QWOA \| \| --- \| \| 1QWYA \| \| 1QX4A \| \| 1QXMA \| \| 1QZMA \| \| 1R0DA \| \| 1R0RI \| \| 1R0UA \| \| 1R17A \| \| 1R1HA \| \| 1R29A \| \| 1R3FA \| \| 1R4PA \| \| 1R4VA \| \| 1R4XA \| \| 1R5LA \| \| 1R5MA \| \| 1R6DA \| \| 1R6JA \| \| 1R6WA \| \| 1R6XA \| \| 1R75A \| \| 1R77A \| \| 1R7AA \| \| 1R7JA \| \| 1R7LA \| \| 1R8SA \| \| 1R9LA \| \| 1R9WA \| \| 1RA0A \| \| 1RCQA \| \| 1REGX \| \| 1REQB \| \| 1RF6A \| \| 1RFYA \| \| 1RG8A \| \| 1RGXA \| \| 1RGZA \| \| 1RI6A \| \| 1RIFA \| \| 1RK6A \| \| 1RK8C \| \| 1RKIA \| \| 1RMGA \| | \| 1RO0A \| \| --- \| \| 1RO7A \| \| 1ROCA \| \| 1RQBA \| \| 1RSSA \| \| 1RT8A \| \| 1RTQA \| \| 1RTTA \| \| 1RU4A \| \| 1RUTX \| \| 1RV9A \| \| 1RW1A \| \| 1RW7A \| \| 1RWHA \| \| 1RWIA \| \| 1RWJA \| \| 1RWRA \| \| 1RWZA \| \| 1RXQA \| \| 1RY9A \| \| 1RYIA \| \| 1RYLA \| \| 1RYOA \| \| 1RYQA \| \| 1RZHH \| \| 1RZHM \| \| 1S0IA \| \| 1S12A \| \| 1S1DA \| \| 1S21A \| \| 1S29A \| \| 1S2XA \| \| 1S3CA \| \| 1S4KA \| \| 1S5AA \| \| 1S5DA \| \| 1S5UA \| \| 1S7IA \| \| 1S7KA \| \| 1S7ZA \| \| 1S99A \| \| 1S9RA \| \| 1S9UA \| \| 1SAUA \| | \| 1SBPA \| \| --- \| \| 1SBXA \| \| 1SBYA \| \| 1SBZA \| \| 1SD4A \| \| 1SDIA \| \| 1SDOA \| \| 1SE8A \| \| 1SEIA \| \| 1SENA \| \| 1SFPA \| \| 1SFSA \| \| 1SG4A \| \| 1SG6A \| \| 1SH8A \| \| 1SJWA \| \| 1SJYA \| \| 1SKZA \| \| 1SL8A \| \| 1SLUA \| \| 1SMBA \| \| 1SMXA \| \| 1SQ9A \| \| 1SQSA \| \| 1SQWA \| \| 1SR4A \| \| 1SR8A \| \| 1SRAA \| \| 1SS4A \| \| 1SSQA \| \| 1STMA \| \| 1SU8A \| \| 1SUMB \| \| 1SURA \| \| 1SUUA \| \| 1SVBA \| \| 1SVFA \| \| 1SVMA \| \| 1SW5A \| \| 1SYYA \| \| 1SZ7A \| \| 1SZHA \| \| 1SZWA \| \| 1T06A \| | \| 1T07A \| \| --- \| \| 1T0BA \| \| 1T0FA \| \| 1T0FC \| \| 1T0HA \| \| 1T0HB \| \| 1T0PB \| \| 1T0TV \| \| 1T15A \| \| 1T1DA \| \| 1T1JA \| \| 1T1UA \| \| 1T1VA \| \| 1T2DA \| \| 1T3TA \| \| 1T3YA \| \| 1T4AA \| \| 1T4BA \| \| 1T5RA \| \| 1T61A \| \| 1T6CA \| \| 1T6EX \| \| 1T6LA \| \| 1T6SA \| \| 1T6UA \| \| 1T7RA \| \| 1T8KA \| \| 1T8TA \| \| 1T92A \| \| 1T9FA \| \| 1TADA \| \| 1TAFA \| \| 1TAFB \| \| 1TBFA \| \| 1TC5A \| \| 1TE2A \| \| 1TFEA \| \| 1TG7A \| \| 1TGRA \| \| 1TH7A \| \| 1THFD \| \| 1THQA \| \| 1TI6B \| \| 1TIFA \| | \| 1TIGA \| \| --- \| \| 1TIQA \| \| 1TJLA \| \| 1TJXA \| \| 1TJYA \| \| 1TKEA \| \| 1TL2A \| \| 1TOAA \| \| 1TP6A \| \| 1TP9A \| \| 1TQGA \| \| 1TQHA \| \| 1TQJA \| \| 1TR0A \| \| 1TS9A \| \| 1TT8A \| \| 1TU1A \| \| 1TU9A \| \| 1TUAA \| \| 1TUKA \| \| 1TUOA \| \| 1TUWA \| \| 1TVXA \| \| 1TWDA \| \| 1TWUA \| \| 1TWYA \| \| 1TXGA \| \| 1TXLA \| \| 1TZPA \| \| 1TZYA \| \| 1TZYB \| \| 1U02A \| \| 1U07A \| \| 1U09A \| \| 1U0SA \| \| 1U14A \| \| 1U2HA \| \| 1U55A \| \| 1U5DA \| \| 1U5KA \| \| 1U5PA \| \| 1U5UA \| \| 1U5VA \| \| 1U5XA \| | \| 1U60A \| \| --- \| \| 1U6ZA \| \| 1U7GA \| \| 1U7IA \| \| 1U7KA \| \| 1U7LA \| \| 1U7PA \| \| 1U84A \| \| 1U8VA \| \| 1U9LA \| \| 1UALA \| \| 1UASA \| \| 1UC8A \| \| 1UCDA \| \| 1UCRA \| \| 1UCSA \| \| 1UD9A \| \| 1UEBA \| \| 1UEKA \| \| 1UFIA \| \| 1UFOA \| \| 1UFYA \| \| 1UG6A \| \| 1UGIA \| \| 1UGXA \| \| 1UI0A \| \| 1UIIA \| \| 1UIXA \| \| 1UJ2A \| \| 1UJ8A \| \| 1UJPA \| \| 1UK8A \| \| 1UKFA \| \| 1UKKA \| \| 1UMGA \| \| 1UNNC \| \| 1UNQA \| \| 1UOYA \| \| 1UPKA \| \| 1UPQA \| \| 1UPTB \| \| 1UQTA \| \| 1URSA \| \| 1US0A \| |
| \| 1US5A \| \| --- \| \| 1USCA \| \| 1USEA \| \| 1USGA \| \| 1USMA \| \| 1USRA \| \| 1UT7A \| \| 1UTEA \| \| 1UTGA \| \| 1UUJA \| \| 1UUQA \| \| 1UUYA \| \| 1UUZA \| \| 1UV7A \| \| 1UW4A \| \| 1UW4B \| \| 1UWCA \| \| 1UWFA \| \| 1UWKA \| \| 1UWWA \| \| 1UX6A \| \| 1UXOA \| \| 1UYLA \| \| 1UZ3A \| \| 1UZEA \| \| 1UZXA \| \| 1V05A \| \| 1V0AA \| \| 1V0WA \| \| 1V2BA \| \| 1V2XA \| \| 1V2ZA \| \| 1V33A \| \| 1V4AA \| \| 1V4PA \| \| 1V54A \| \| 1V54B \| \| 1V54C \| \| 1V54D \| \| 1V54E \| \| 1V54F \| \| 1V54G \| \| 1V54H \| \| 1V54I \| | \| 1V54J \| \| --- \| \| 1V54K \| \| 1V54L \| \| 1V54M \| \| 1V5IB \| \| 1V5VA \| \| 1V6PA \| \| 1V6SA \| \| 1V6TA \| \| 1V74A \| \| 1V74B \| \| 1V77A \| \| 1V7WA \| \| 1V84A \| \| 1V8HA \| \| 1V96A \| \| 1V9FA \| \| 1V9MA \| \| 1V9YA \| \| 1VAJA \| \| 1VBKA \| \| 1VBWA \| \| 1VCAA \| \| 1VCCA \| \| 1VD6A \| \| 1VDKA \| \| 1VDWA \| \| 1VE1A \| \| 1VE2A \| \| 1VE4A \| \| 1VEFA \| \| 1VFYA \| \| 1VGJA \| \| 1VGYA \| \| 1VH4A \| \| 1VH5A \| \| 1VHNA \| \| 1VHTA \| \| 1VHUA \| \| 1VHWA \| \| 1VI0A \| \| 1VI4A \| \| 1VI6A \| \| 1VI9A \| | \| 1VJFA \| \| --- \| \| 1VJLA \| \| 1VJNA \| \| 1VJUA \| \| 1VJVA \| \| 1VK1A \| \| 1VKEA \| \| 1VKFA \| \| 1VKHA \| \| 1VKKA \| \| 1VKMA \| \| 1VKNA \| \| 1VKYA \| \| 1VL1A \| \| 1VL7A \| \| 1VLPA \| \| 1VLSA \| \| 1VLYA \| \| 1VMBA \| \| 1VMEA \| \| 1VMGA \| \| 1VMHA \| \| 1VP8A \| \| 1VPBA \| \| 1VPRA \| \| 1VPSA \| \| 1VPTA \| \| 1VQSA \| \| 1VR7A \| \| 1VRAA \| \| 1VRAB \| \| 1VRMA \| \| 1VSRA \| \| 1VYBA \| \| 1VYIA \| \| 1VYKA \| \| 1VYRA \| \| 1VZIA \| \| 1VZMA \| \| 1VZYA \| \| 1W07A \| \| 1W0HA \| \| 1W0NA \| \| 1W1HA \| | \| 1W23A \| \| --- \| \| 1W2FA \| \| 1W2WA \| \| 1W2WB \| \| 1W2YA \| \| 1W3IA \| \| 1W4SA \| \| 1W4XA \| \| 1W53A \| \| 1W5QA \| \| 1W5RA \| \| 1W66A \| \| 1W6GA \| \| 1W6SA \| \| 1W6SB \| \| 1W78A \| \| 1W7CA \| \| 1W8KA \| \| 1W8SA \| \| 1W94A \| \| 1W96A \| \| 1W99A \| \| 1W9HA \| \| 1WA5B \| \| 1WA5C \| \| 1WAAA \| \| 1WAPA \| \| 1WB4A \| \| 1WBAA \| \| 1WBEA \| \| 1WBHA \| \| 1WC1A \| \| 1WC2A \| \| 1WCWA \| \| 1WD3A \| \| 1WDDA \| \| 1WDDS \| \| 1WDJA \| \| 1WDPA \| \| 1WEHA \| \| 1WERA \| \| 1WG8A \| \| 1WHIA \| \| 1WHSA \| | \| 1WHZA \| \| --- \| \| 1WIWA \| \| 1WJ9A \| \| 1WJXA \| \| 1WL8A \| \| 1WLEA \| \| 1WLGA \| \| 1WLJA \| \| 1WLUA \| \| 1WLZA \| \| 1WMHA \| \| 1WMHB \| \| 1WMWA \| \| 1WMXA \| \| 1WN2A \| \| 1WNAA \| \| 1WNYA \| \| 1WO8A \| \| 1WOCA \| \| 1WOJA \| \| 1WOLA \| \| 1WOQA \| \| 1WOUA \| \| 1WPAA \| \| 1WPBA \| \| 1WPNA \| \| 1WPVA \| \| 1WQ6A \| \| 1WQJB \| \| 1WRAA \| \| 1WRDA \| \| 1WS8A \| \| 1WT6A \| \| 1WTJA \| \| 1WUBA \| \| 1WUIL \| \| 1WUIS \| \| 1WURA \| \| 1WV3A \| \| 1WVFA \| \| 1WVGA \| \| 1WVHA \| \| 1WVQA \| \| 1WWIA \| | \| 1WWJA \| \| --- \| \| 1WWZA \| \| 1WXCA \| \| 1WXCB \| \| 1WY2A \| \| 1WZ3A \| \| 1WZDA \| \| 1WZUA \| \| 1X0TA \| \| 1X1NA \| \| 1X2IA \| \| 1X38A \| \| 1X54A \| \| 1X6IA \| \| 1X6OA \| \| 1X6ZA \| \| 1X7DA \| \| 1X8BA \| \| 1X8QA \| \| 1X91A \| \| 1X9DA \| \| 1X9IA \| \| 1XAKA \| \| 1XAUA \| \| 1XBIA \| \| 1XCLA \| \| 1XCRA \| \| 1XD3A \| \| 1XDNA \| \| 1XE7A \| \| 1XEWX \| \| 1XEWY \| \| 1XFFA \| \| 1XFKA \| \| 1XFSA \| \| 1XG0A \| \| 1XG0C \| \| 1XG4A \| \| 1XGKA \| \| 1XHNA \| \| 1XIWA \| \| 1XIZA \| \| 1XJJA \| \| 1XJUA \| | \| 1XKIA \| \| --- \| \| 1XKPA \| \| 1XKPB \| \| 1XKPC \| \| 1XKRA \| \| 1XKWA \| \| 1XLQA \| \| 1XLYA \| \| 1XMKA \| \| 1XMTA \| \| 1XODA \| \| 1XOVA \| \| 1XPPA \| \| 1XQAA \| \| 1XQOA \| \| 1XRUA \| \| 1XS0A \| \| 1XS5A \| \| 1XSVA \| \| 1XSZA \| \| 1XTTA \| \| 1XU1R \| \| 1XUBA \| \| 1XV2A \| \| 1XV5A \| \| 1XVHA \| \| 1XW3A \| \| 1XWVA \| \| 1XWWA \| \| 1XX1A \| \| 1XZZA \| \| 1Y08A \| \| 1Y0KA \| \| 1Y0NA \| \| 1Y0UA \| \| 1Y12A \| \| 1Y37A \| \| 1Y43B \| \| 1Y4WA \| \| 1Y5HA \| \| 1Y5IC \| \| 1Y60A \| \| 1Y66A \| \| 1Y6XA \| |
| \| 1Y6ZA \| \| --- \| \| 1Y71A \| \| 1Y7PA \| \| 1Y7TA \| \| 1Y8AA \| \| 1Y93A \| \| 1Y9LA \| \| 1YARA \| \| 1YARO \| \| 1YB0A \| \| 1YB3A \| \| 1YBKA \| \| 1YC5A \| \| 1YCDA \| \| 1YD0A \| \| 1YD9A \| \| 1YDGA \| \| 1YDIA \| \| 1YDYA \| \| 1YE8A \| \| 1YFQA \| \| 1YFUA \| \| 1YG9A \| \| 1YGAA \| \| 1YGTA \| \| 1YHTA \| \| 1YI9A \| \| 1YJ7A \| \| 1YKIA \| \| 1YLIA \| \| 1YLKA \| \| 1YLLA \| \| 1YLMA \| \| 1YLXA \| \| 1YM3A \| \| 1YMTA \| \| 1YN3A \| \| 1YNFA \| \| 1YNPA \| \| 1YO3A \| \| 1YOCA \| \| 1YOZA \| \| 1YPYA \| \| 1YQ5A \| | \| 1YQEA \| \| --- \| \| 1YQGA \| \| 1YQHA \| \| 1YQSA \| \| 1YRKA \| \| 1YRRA \| \| 1YSRA \| \| 1YT3A \| \| 1YT8A \| \| 1YTLA \| \| 1YU0A \| \| 1YU5X \| \| 1YUMA \| \| 1YW4A \| \| 1YWFA \| \| 1YX1A \| \| 1YZ1A \| \| 1YZXA \| \| 1Z0JB \| \| 1Z0MA \| \| 1Z0PA \| \| 1Z0WA \| \| 1Z1YA \| \| 1Z2NX \| \| 1Z2UA \| \| 1Z2WA \| \| 1Z3EA \| \| 1Z3EB \| \| 1Z3XA \| \| 1Z5ZA \| \| 1Z67A \| \| 1Z6MA \| \| 1Z6OA \| \| 1Z6OM \| \| 1Z70X \| \| 1Z72A \| \| 1Z96A \| \| 1Z9LA \| \| 1ZA0A \| \| 1ZARA \| \| 1ZAVA \| \| 1ZB1A \| \| 1ZC3B \| \| 1ZCEA \| | \| 1ZD0A \| \| --- \| \| 1ZD7A \| \| 1ZD8A \| \| 1ZDYA \| \| 1ZELA \| \| 1ZGKA \| \| 1ZHSA \| \| 1ZHVA \| \| 1ZHXA \| \| 1ZI8A \| \| 1ZJAA \| \| 1ZJCA \| \| 1ZK4A \| \| 1ZK5A \| \| 1ZKEA \| \| 1ZL0A \| \| 1ZLDA \| \| 1ZMAA \| \| 1ZMTA \| \| 1ZN6A \| \| 1ZPSA \| \| 1ZS9A \| \| 1ZSQA \| \| 1ZT3A \| \| 1ZTDA \| \| 1ZUUA \| \| 1ZV1A \| \| 1ZVAA \| \| 1ZVTA \| \| 1ZX8A \| \| 1ZXXA \| \| 1ZY7A \| \| 1ZZ1A \| \| 1ZZKA \| \| 256BA \| \| 2A14A \| \| 2A1HA \| \| 2A1IA \| \| 2A1KA \| \| 2A26A \| \| 2A2KA \| \| 2A2MA \| \| 2A35A \| \| 2A3NA \| | \| 2A40B \| \| --- \| \| 2A4XA \| \| 2A65A \| \| 2A6SA \| \| 2A6ZA \| \| 2A72A \| \| 2A7BA \| \| 2A8YA \| \| 2A9DA \| \| 2A9IA \| \| 2A9SA \| \| 2ABKA \| \| 2ABSA \| \| 2AEBA \| \| 2AEUA \| \| 2AFWA \| \| 2AG4A \| \| 2AGKA \| \| 2AHFA \| \| 2AIBA \| \| 2AJ6A \| \| 2AJ7A \| \| 2AKAB \| \| 2AKZA \| \| 2AMHA \| \| 2AMLA \| \| 2ANXA \| \| 2AO9A \| \| 2AP3A \| \| 2APJA \| \| 2APOB \| \| 2AQ6A \| \| 2ASKA \| \| 2ATZA \| \| 2AU7A \| \| 2AUWA \| \| 2AVDA \| \| 2AVTA \| \| 2AXCA \| \| 2AXOA \| \| 2AXWA \| \| 2AYDA \| \| 2AZ4A \| \| 2B06A \| | \| 2B0AA \| \| --- \| \| 2B0TA \| \| 2B0VA \| \| 2B1YA \| \| 2B3GA \| \| 2B4HA \| \| 2B4VA \| \| 2B4WA \| \| 2B50A \| \| 2B5AA \| \| 2B5GA \| \| 2B5WA \| \| 2B7KA \| \| 2B82A \| \| 2B8IA \| \| 2B8MA \| \| 2B97A \| \| 2B9DA \| \| 2B9EA \| \| 2BA2A \| \| 2BAYA \| \| 2BB6A \| \| 2BBAA \| \| 2BBEA \| \| 2BBRA \| \| 2BCMA \| \| 2BDRA \| \| 2BEKA \| \| 2BEMA \| \| 2BF6A \| \| 2BFDA \| \| 2BFDB \| \| 2BG1A \| \| 2BG5A \| \| 2BGIA \| \| 2BHUA \| \| 2BI0A \| \| 2BJFA \| \| 2BJIA \| \| 2BJKA \| \| 2BJNA \| \| 2BJQA \| \| 2BJVA \| \| 2BKFA \| | \| 2BKMA \| \| --- \| \| 2BKXA \| \| 2BKYA \| \| 2BL0A \| \| 2BL8A \| \| 2BLNA \| \| 2BM5A \| \| 2BMOA \| \| 2BMOB \| \| 2BMWA \| \| 2BNLA \| \| 2BNMA \| \| 2BO9B \| \| 2BOGX \| \| 2BONA \| \| 2BOUA \| \| 2BPTA \| \| 2BRFA \| \| 2BRJA \| \| 2BRYA \| \| 2BS2B \| \| 2BS2C \| \| 2BSJA \| \| 2BT9A \| \| 2BTIA \| \| 2BU3A \| \| 2BUEA \| \| 2BW3B \| \| 2BWFA \| \| 2BWQA \| \| 2BWRA \| \| 2BZ1A \| \| 2BZVA \| \| 2C0GA \| \| 2C0HA \| \| 2C0NA \| \| 2C1VA \| \| 2C2IA \| \| 2C2QA \| \| 2C2UA \| \| 2C3VA \| \| 2C4XA \| \| 2C5AA \| \| 2C5LC \| | \| 2C61A \| \| --- \| \| 2C6QA \| \| 2C71A \| \| 2C78A \| \| 2C82A \| \| 2C8EE \| \| 2C8MA \| \| 2C92A \| \| 2C9WA \| \| 2CARA \| \| 2CAYA \| \| 2CB2A \| \| 2CB8A \| \| 2CBZA \| \| 2CC6A \| \| 2CCHB \| \| 2CCMA \| \| 2CCQA \| \| 2CCVA \| \| 2CDUA \| \| 2CE2X \| \| 2CFUA \| \| 2CG7A \| \| 2CH5A \| \| 2CHCA \| \| 2CHHA \| \| 2CHOA \| \| 2CI1A \| \| 2CIBA \| \| 2CIUA \| \| 2CIWA \| \| 2CJ4A \| \| 2CJSC \| \| 2CJTA \| \| 2CKKA \| \| 2CKLB \| \| 2CKXA \| \| 2CM4A \| \| 2CMGA \| \| 2CMPA \| \| 2CN3A \| \| 2CNQA \| \| 2CO3A \| \| 2COVD \| |
| \| 2CPGA \| \| --- \| \| 2CS7A \| \| 2CU3A \| \| 2CUAA \| \| 2CVDA \| \| 2CVEA \| \| 2CW9A \| \| 2CWRA \| \| 2CWSA \| \| 2CWYA \| \| 2CWZA \| \| 2CX1A \| \| 2CX7A \| \| 2CXAA \| \| 2CXCA \| \| 2CXHA \| \| 2CXIA \| \| 2CXKA \| \| 2CXNA \| \| 2CXYA \| \| 2CY5A \| \| 2CYGA \| \| 2CYJA \| \| 2CZLA \| \| 2CZSA \| \| 2CZVC \| \| 2D0OA \| \| 2D1LA \| \| 2D1SA \| \| 2D28C \| \| 2D29A \| \| 2D3DA \| \| 2D48A \| \| 2D4PA \| \| 2D4XA \| \| 2D59A \| \| 2D5BA \| \| 2D5FA \| \| 2D5MA \| \| 2D5WA \| \| 2D68A \| \| 2D7CC \| \| 2D7VA \| \| 2D80A \| | \| 2D8DA \| \| --- \| \| 2DB7A \| \| 2DBNA \| \| 2DBYA \| \| 2DC4A \| \| 2DCFA \| \| 2DDRA \| \| 2DDXA \| \| 2DE3A \| \| 2DE6A \| \| 2DEJA \| \| 2DG1A \| \| 2DG5A \| \| 2DGKA \| \| 2DJFA \| \| 2DJIA \| \| 2DKHA \| \| 2DKJA \| \| 2DKOA \| \| 2DKOB \| \| 2DLBA \| \| 2DM9A \| \| 2DOKA \| \| 2DP9A \| \| 2DPFA \| \| 2DPLA \| \| 2DPMA \| \| 2DQAA \| \| 2DQLA \| \| 2DQWA \| \| 2DS2B \| \| 2DS5A \| \| 2DSJA \| \| 2DSKA \| \| 2DSTA \| \| 2DSXA \| \| 2DSYA \| \| 2DT8A \| \| 2DTJA \| \| 2DVMA \| \| 2DVTA \| \| 2DWKA \| \| 2DWUA \| \| 2DXAA \| | \| 2DXQA \| \| --- \| \| 2DXUA \| \| 2DY0A \| \| 2DYIA \| \| 2DYJA \| \| 2DYOA \| \| 2DYOB \| \| 2E01A \| \| 2E11A \| \| 2E1FA \| \| 2E1VA \| \| 2E1ZA \| \| 2E2DC \| \| 2E2OA \| \| 2E3HA \| \| 2E3NA \| \| 2E4MC \| \| 2E4TA \| \| 2E56A \| \| 2E5FA \| \| 2E5YA \| \| 2E6FA \| \| 2E6MA \| \| 2E6XA \| \| 2E7VA \| \| 2E7ZA \| \| 2E85A \| \| 2E8BA \| \| 2E8EA \| \| 2E8VA \| \| 2EABA \| \| 2EAQA \| \| 2EB4A \| \| 2EBEA \| \| 2EBNA \| \| 2EBOA \| \| 2ECEA \| \| 2EFJA \| \| 2EFVA \| \| 2EGVA \| \| 2EGZA \| \| 2EH3A \| \| 2EHPA \| \| 2EHZA \| | \| 2EI9A \| \| --- \| \| 2EJAA \| \| 2EJXA \| \| 2EK0A \| \| 2EKLA \| \| 2ELCA \| \| 2ENDA \| \| 2ENGA \| \| 2EPLX \| \| 2EQ7C \| \| 2ERBA \| \| 2ERFA \| \| 2ERLA \| \| 2ERVA \| \| 2ES9A \| \| 2ESSA \| \| 2ETJA \| \| 2ETVA \| \| 2ETXA \| \| 2EV1A \| \| 2EVBA \| \| 2EW0A \| \| 2EWTA \| \| 2EX0A \| \| 2EX2A \| \| 2EX4A \| \| 2EZ2A \| \| 2F01A \| \| 2F1FA \| \| 2F1KA \| \| 2F1NA \| \| 2F22A \| \| 2F23A \| \| 2F2EA \| \| 2F2HA \| \| 2F46A \| \| 2F4MA \| \| 2F4MB \| \| 2F5GA \| \| 2F5TX \| \| 2F5XA \| \| 2F60K \| \| 2F62A \| \| 2F6EA \| | \| 2F6UA \| \| --- \| \| 2F7VA \| \| 2F9FA \| \| 2F9HA \| \| 2FA1A \| \| 2FA8A \| \| 2FAOA \| \| 2FB5A \| \| 2FB6A \| \| 2FBAA \| \| 2FBLA \| \| 2FCJA \| \| 2FCKA \| \| 2FCLA \| \| 2FCOA \| \| 2FCTA \| \| 2FCWA \| \| 2FCWB \| \| 2FD4A \| \| 2FD6U \| \| 2FDNA \| \| 2FE3A \| \| 2FE8A \| \| 2FEAA \| \| 2FEFA \| \| 2FFUA \| \| 2FG1A \| \| 2FGQX \| \| 2FH1A \| \| 2FHFA \| \| 2FHPA \| \| 2FHZA \| \| 2FHZB \| \| 2FI1A \| \| 2FI9A \| \| 2FIPA \| \| 2FIUA \| \| 2FJ8A \| \| 2FJRA \| \| 2FKKA \| \| 2FL7A \| \| 2FLHA \| \| 2FM9A \| \| 2FMAA \| | \| 2FNAA \| \| --- \| \| 2FOJA \| \| 2FOMA \| \| 2FOMB \| \| 2FOZA \| \| 2FP1A \| \| 2FPHX \| \| 2FQ3A \| \| 2FQXA \| \| 2FR5A \| \| 2FREA \| \| 2FRGP \| \| 2FSHA \| \| 2FSQA \| \| 2FSRA \| \| 2FSUA \| \| 2FT0A \| \| 2FTRA \| \| 2FTXA \| \| 2FTXB \| \| 2FUEA \| \| 2FUFA \| \| 2FULA \| \| 2FUPA \| \| 2FURA \| \| 2FVYA \| \| 2FWHA \| \| 2FWTA \| \| 2FXUA \| \| 2FY7A \| \| 2FYGA \| \| 2FYQA \| \| 2FZSA \| \| 2G0WA \| \| 2G2CA \| \| 2G30A \| \| 2G3RA \| \| 2G3WA \| \| 2G40A \| \| 2G45A \| \| 2G5FA \| \| 2G5GX \| \| 2G64A \| \| 2G7BA \| | \| 2G7IA \| \| --- \| \| 2G7OA \| \| 2G7SA \| \| 2G82A \| \| 2G84A \| \| 2G8SA \| \| 2G9WA \| \| 2GA1A \| \| 2GAGB \| \| 2GAGC \| \| 2GAGD \| \| 2GAKA \| \| 2GAUA \| \| 2GAXA \| \| 2GB4A \| \| 2GCIA \| \| 2GDMA \| \| 2GDQA \| \| 2GF6A \| \| 2GFFA \| \| 2GGCA \| \| 2GHSA \| \| 2GHTA \| \| 2GIAA \| \| 2GIBA \| \| 2GIYA \| \| 2GJ3A \| \| 2GJ4A \| \| 2GJLA \| \| 2GK4A \| \| 2GKEA \| \| 2GKGA \| \| 2GKPA \| \| 2GLZA \| \| 2GMQA \| \| 2GNOA \| \| 2GNPA \| \| 2GOMA \| \| 2GPEA \| \| 2GPIA \| \| 2GR8A \| \| 2GRCA \| \| 2GRRB \| \| 2GS5A \| |
| \| 2GS8A \| \| --- \| \| 2GSOA \| \| 2GSVA \| \| 2GT1A \| \| 2GTRA \| \| 2GU3A \| \| 2GU9A \| \| 2GUDA \| \| 2GUFA \| \| 2GUIA \| \| 2GUKA \| \| 2GUYA \| \| 2GUZB \| \| 2GVIA \| \| 2GVKA \| \| 2GWMA \| \| 2GWNA \| \| 2GX5A \| \| 2GXQA \| \| 2GYQA \| \| 2GZ4A \| \| 2GZ6A \| \| 2GZBA \| \| 2GZQA \| \| 2GZSA \| \| 2H00A \| \| 2H1CA \| \| 2H1TA \| \| 2H1VA \| \| 2H26A \| \| 2H2ZA \| \| 2H5OA \| \| 2H62C \| \| 2H6FA \| \| 2H6FB \| \| 2H7MA \| \| 2H7ZA \| \| 2H88A \| \| 2H88C \| \| 2H88D \| \| 2H8EA \| \| 2H8GA \| \| 2H98A \| \| 2H9AB \| | \| 2H9DA \| \| --- \| \| 2HA8A \| \| 2HAIA \| \| 2HALA \| \| 2HBAA \| \| 2HBTA \| \| 2HBWA \| \| 2HC1A \| \| 2HC8A \| \| 2HCMA \| \| 2HD9A \| \| 2HDWA \| \| 2HEUA \| \| 2HEWF \| \| 2HF1A \| \| 2HFKA \| \| 2HFNA \| \| 2HFTA \| \| 2HHCA \| \| 2HHPA \| \| 2HI0A \| \| 2HIMA \| \| 2HINA \| \| 2HIYA \| \| 2HJEA \| \| 2HJNA \| \| 2HK0A \| \| 2HKVA \| \| 2HL7A \| \| 2HLJA \| \| 2HLRA \| \| 2HLYA \| \| 2HLZA \| \| 2HNGA \| \| 2HNUA \| \| 2HO3A \| \| 2HOXA \| \| 2HP0A \| \| 2HP7A \| \| 2HQ2A \| \| 2HQ7A \| \| 2HQLA \| \| 2HQSA \| \| 2HQSC \| | \| 2HQTA \| \| --- \| \| 2HQXA \| \| 2HQYA \| \| 2HRAA \| \| 2HS1A \| \| 2HSBA \| \| 2HSIA \| \| 2HTSA \| \| 2HU9A \| \| 2HUEB \| \| 2HUEC \| \| 2HUHA \| \| 2HVWA \| \| 2HW2A \| \| 2HX0A \| \| 2HX5A \| \| 2HY5A \| \| 2HY5C \| \| 2HY7A \| \| 2HYKA \| \| 2HZCA \| \| 2HZLA \| \| 2I0KA \| \| 2I2CA \| \| 2I33A \| \| 2I49A \| \| 2I4LA \| \| 2I53A \| \| 2I5HA \| \| 2I5IA \| \| 2I5UA \| \| 2I5VO \| \| 2I6CA \| \| 2I6HA \| \| 2I74A \| \| 2I7AA \| \| 2I7DA \| \| 2I7GA \| \| 2I7NA \| \| 2I8DA \| \| 2I8GA \| \| 2I8TA \| \| 2I9CA \| \| 2I9FA \| | \| 2I9IA \| \| --- \| \| 2I9WA \| \| 2I9XA \| \| 2IA1A \| \| 2IA7A \| \| 2IAYA \| \| 2IB0A \| \| 2IBAA \| \| 2IBLA \| \| 2IBNA \| \| 2IC2A \| \| 2IC6A \| \| 2ICHA \| \| 2ICUA \| \| 2ICYA \| \| 2ID4A \| \| 2IDLA \| \| 2IF6A \| \| 2IGIA \| \| 2IGPA \| \| 2IGXA \| \| 2II2A \| \| 2IIAA \| \| 2IIHA \| \| 2IJ2A \| \| 2IKSA \| \| 2ILKA \| \| 2ILRA \| \| 2IM8A \| \| 2IM9A \| \| 2IMFA \| \| 2IMHA \| \| 2IMJA \| \| 2IMQX \| \| 2IMRA \| \| 2IMSA \| \| 2IN0A \| \| 2IN3A \| \| 2INCB \| \| 2INUA \| \| 2INWA \| \| 2IP1A \| \| 2IP6A \| \| 2IPIA \| | \| 2IQYA \| \| --- \| \| 2ISBA \| \| 2IT2A \| \| 2IT9A \| \| 2ITEA \| \| 2IU5A \| \| 2IUWA \| \| 2IVFC \| \| 2IVNA \| \| 2IVYA \| \| 2IW1A \| \| 2IWAA \| \| 2IWBA \| \| 2IWRA \| \| 2IXDA \| \| 2IXMA \| \| 2IXSA \| \| 2IY2A \| \| 2IYFA \| \| 2IYVA \| \| 2IZ6A \| \| 2IZRA \| \| 2IZXA \| \| 2J0AA \| \| 2J1PA \| \| 2J1VA \| \| 2J43A \| \| 2J5GA \| \| 2J5YA \| \| 2J66A \| \| 2J6AA \| \| 2J6BA \| \| 2J6GA \| \| 2J6LA \| \| 2J6VA \| \| 2J73A \| \| 2J7JA \| \| 2J7QA \| \| 2J7UA \| \| 2J8BA \| \| 2J8KA \| \| 2J8WA \| \| 2J97A \| \| 2J9CA \| | \| 2J9OA \| \| --- \| \| 2J9UB \| \| 2J9WA \| \| 2JC5A \| \| 2JC9A \| \| 2JCBA \| \| 2JCQA \| \| 2JDCA \| \| 2JDID \| \| 2JDIG \| \| 2JDIH \| \| 2JDII \| \| 2JDJA \| \| 2JE3A \| \| 2JE6A \| \| 2JE6B \| \| 2JE6I \| \| 2JE8A \| \| 2JEKA \| \| 2JEPA \| \| 2JFRA \| \| 2JG0A \| \| 2JGBA \| \| 2JGPA \| \| 2JH1A \| \| 2JH3A \| \| 2JHFA \| \| 2JHNA \| \| 2JK9A \| \| 2JKGA \| \| 2JKHL \| \| 2JLIA \| \| 2JLQA \| \| 2LISA \| \| 2MBRA \| \| 2MCMA \| \| 2MHRA \| \| 2NL9A \| \| 2NLRA \| \| 2NLVA \| \| 2NMLA \| \| 2NNUA \| \| 2NOOA \| \| 2NP5A \| | \| 2NPNA \| \| --- \| \| 2NPTA \| \| 2NPTB \| \| 2NQ5A \| \| 2NQLA \| \| 2NQWA \| \| 2NR5A \| \| 2NR7A \| \| 2NRKA \| \| 2NRLA \| \| 2NRRA \| \| 2NS9A \| \| 2NSAA \| \| 2NSZA \| \| 2NT0A \| \| 2NTPA \| \| 2NUHA \| \| 2NUJA \| \| 2NVHA \| \| 2NVOA \| \| 2NW2A \| \| 2NW8A \| \| 2NWFA \| \| 2NWHA \| \| 2NX2A \| \| 2NX4A \| \| 2NXFA \| \| 2NXVA \| \| 2NY1A \| \| 2NYDA \| \| 2NYIA \| \| 2NZ7A \| \| 2NZCA \| \| 2NZXA \| \| 2O02A \| \| 2O0AA \| \| 2O0BA \| \| 2O0MA \| \| 2O0QA \| \| 2O1KA \| \| 2O1MA \| \| 2O1QA \| \| 2O2KA \| \| 2O2XA \| |
| \| 2O30A \| \| --- \| \| 2O34A \| \| 2O36A \| \| 2O38A \| \| 2O4TA \| \| 2O4UX \| \| 2O4VA \| \| 2O5HA \| \| 2O5UA \| \| 2O62A \| \| 2O6FA \| \| 2O6PA \| \| 2O6SA \| \| 2O70A \| \| 2O71A \| \| 2O7AA \| \| 2O7IA \| \| 2O7MA \| \| 2O8MA \| \| 2O8PA \| \| 2O8QA \| \| 2O90A \| \| 2O95A \| \| 2O99A \| \| 2O9CA \| \| 2O9SA \| \| 2O9UX \| \| 2OA9A \| \| 2OAFA \| \| 2OB3A \| \| 2OB5A \| \| 2OBPA \| \| 2OC5A \| \| 2OCTA \| \| 2OD4A \| \| 2OD5A \| \| 2OD6A \| \| 2ODAA \| \| 2ODFA \| \| 2ODHA \| \| 2ODKA \| \| 2OEBA \| \| 2OEEA \| \| 2OEZA \| | \| 2OF3A \| \| --- \| \| 2OFCA \| \| 2OFKA \| \| 2OFZA \| \| 2OG4A \| \| 2OGBA \| \| 2OGFA \| \| 2OGXA \| \| 2OH3A \| \| 2OH5A \| \| 2OHWA \| \| 2OITA \| \| 2OIWA \| \| 2OIXA \| \| 2OIZA \| \| 2OIZD \| \| 2OJ5A \| \| 2OJPA \| \| 2OKFA \| \| 2OKMA \| \| 2OKTA \| \| 2OKUA \| \| 2OLMA \| \| 2OLRA \| \| 2OLTA \| \| 2OMLA \| \| 2OMZB \| \| 2OO3A \| \| 2OOAA \| \| 2OOKA \| \| 2OPCA \| \| 2OPLA \| \| 2OQ0A \| \| 2OQBA \| \| 2OQKA \| \| 2OQQA \| \| 2OQZA \| \| 2ORWA \| \| 2OS0A \| \| 2OSOA \| \| 2OSXA \| \| 2OTMA \| \| 2OU1A \| \| 2OU3A \| | \| 2OU5A \| \| --- \| \| 2OU6A \| \| 2OUFA \| \| 2OV0A \| \| 2OVGA \| \| 2OVJA \| \| 2OVSA \| \| 2OWAA \| \| 2OX6A \| \| 2OX7A \| \| 2OXGB \| \| 2OXLA \| \| 2OXOA \| \| 2OY9A \| \| 2OYAA \| \| 2OYOA \| \| 2OYZA \| \| 2OZJA \| \| 2OZNA \| \| 2OZTA \| \| 2P02A \| \| 2P08A \| \| 2P09A \| \| 2P0AA \| \| 2P0NA \| \| 2P0SA \| \| 2P0WA \| \| 2P12A \| \| 2P14A \| \| 2P1MA \| \| 2P1MB \| \| 2P26A \| \| 2P2SA \| \| 2P38A \| \| 2P3PA \| \| 2P3YA \| \| 2P45B \| \| 2P4FA \| \| 2P4HX \| \| 2P4OA \| \| 2P4PA \| \| 2P51A \| \| 2P58A \| \| 2P58B \| | \| 2P58C \| \| --- \| \| 2P5KA \| \| 2P65A \| \| 2P67A \| \| 2P6PA \| \| 2P6VA \| \| 2P6WA \| \| 2P8GA \| \| 2P8IA \| \| 2P97A \| \| 2P9BA \| \| 2P9WA \| \| 2P9XA \| \| 2PA7A \| \| 2PA8D \| \| 2PA8L \| \| 2PAGA \| \| 2PBDP \| \| 2PBDV \| \| 2PBIB \| \| 2PBKA \| \| 2PC1A \| \| 2PC8A \| \| 2PD1A \| \| 2PEFA \| \| 2PEQA \| \| 2PFIA \| \| 2PFZA \| \| 2PGEA \| \| 2PGNA \| \| 2PH0A \| \| 2PHNA \| \| 2PIEA \| \| 2PK8A \| \| 2PKEA \| \| 2PKFA \| \| 2PKHA \| \| 2PMAA \| \| 2PMRA \| \| 2PMUA \| \| 2PN1A \| \| 2PN6A \| \| 2PNDA \| \| 2PNEA \| | \| 2PNQA \| \| --- \| \| 2PNWA \| \| 2PO4A \| \| 2POFA \| \| 2PORA \| \| 2PPNA \| \| 2PPQA \| \| 2PPVA \| \| 2PPXA \| \| 2PQ7A \| \| 2PQ8A \| \| 2PQRA \| \| 2PQRC \| \| 2PQXA \| \| 2PR7A \| \| 2PRSA \| \| 2PRVA \| \| 2PRXA \| \| 2PS1A \| \| 2PSTX \| \| 2PTHA \| \| 2PTMA \| \| 2PTRA \| \| 2PTTB \| \| 2PU3A \| \| 2PU9A \| \| 2PU9B \| \| 2PUYA \| \| 2PV2A \| \| 2PV4A \| \| 2PVBA \| \| 2PVQA \| \| 2PW0A \| \| 2PWWA \| \| 2PXXA \| \| 2PYQA \| \| 2PYWA \| \| 2PYXA \| \| 2Q03A \| \| 2Q09A \| \| 2Q0OA \| \| 2Q0SA \| \| 2Q0ZX \| \| 2Q12A \| | \| 2Q2GA \| \| --- \| \| 2Q35A \| \| 2Q3TA \| \| 2Q40A \| \| 2Q43A \| \| 2Q46A \| \| 2Q48A \| \| 2Q4FA \| \| 2Q4MA \| \| 2Q4NA \| \| 2Q4ZA \| \| 2Q5CA \| \| 2Q6KA \| \| 2Q6QA \| \| 2Q79A \| \| 2Q7SA \| \| 2Q7WA \| \| 2Q82A \| \| 2Q88A \| \| 2Q8GA \| \| 2Q8KA \| \| 2Q8PA \| \| 2Q9FA \| \| 2Q9KA \| \| 2Q9OA \| \| 2Q9RA \| \| 2QACA \| \| 2QAHA \| \| 2QAPA \| \| 2QB7A \| \| 2QC1B \| \| 2QCKA \| \| 2QCPX \| \| 2QCVA \| \| 2QDJA \| \| 2QE8A \| \| 2QEDA \| \| 2QEEA \| \| 2QEUA \| \| 2QF4A \| \| 2QFAA \| \| 2QFAB \| \| 2QFAC \| \| 2QFEA \| | \| 2QFFA \| \| --- \| \| 2QGUA \| \| 2QGYA \| \| 2QH9A \| \| 2QHFA \| \| 2QHKA \| \| 2QHOB \| \| 2QHPA \| \| 2QHQA \| \| 2QIAA \| \| 2QIKA \| \| 2QIMA \| \| 2QIPA \| \| 2QIYC \| \| 2QJLA \| \| 2QJVA \| \| 2QJWA \| \| 2QJZA \| \| 2QK1A \| \| 2QKDA \| \| 2QKFA \| \| 2QKHA \| \| 2QKHB \| \| 2QKPA \| \| 2QL8A \| \| 2QLTA \| \| 2QLWA \| \| 2QMAA \| \| 2QMCB \| \| 2QMJA \| \| 2QMLA \| \| 2QMMA \| \| 2QNGA \| \| 2QNIA \| \| 2QNLA \| \| 2QOLA \| \| 2QORA \| \| 2QP2A \| \| 2QPXA \| \| 2QQ4A \| \| 2QQ9A \| \| 2QR6A \| \| 2QRLA \| \| 2QRUA \| |
| \| 2QSAA \| \| --- \| \| 2QSBA \| \| 2QSIA \| \| 2QSKA \| \| 2QSQA \| \| 2QSWA \| \| 2QSXA \| \| 2QT1A \| \| 2QT7A \| \| 2QTCA \| \| 2QTDA \| \| 2QTSA \| \| 2QTWA \| \| 2QUDA \| \| 2QUOA \| \| 2QUPA \| \| 2QV5A \| \| 2QV6A \| \| 2QV8A \| \| 2QVOA \| \| 2QW5A \| \| 2QWOB \| \| 2QWUA \| \| 2QWXA \| \| 2QXFA \| \| 2QY1A \| \| 2QY6A \| \| 2QYCA \| \| 2QYWA \| \| 2QZCA \| \| 2QZQA \| \| 2R01A \| \| 2R0XA \| \| 2R16A \| \| 2R1IA \| \| 2R25A \| \| 2R2AA \| \| 2R2CA \| \| 2R2YA \| \| 2R2ZA \| \| 2R31A \| \| 2R47A \| \| 2R4FA \| \| 2R4GA \| | \| 2R4IA \| \| --- \| \| 2R4QA \| \| 2R5DA \| \| 2R5OA \| \| 2R5UA \| \| 2R6JA \| \| 2R6OA \| \| 2R6UA \| \| 2R6ZA \| \| 2R751 \| \| 2R7DA \| \| 2R7GA \| \| 2R85A \| \| 2R8EA \| \| 2R8OA \| \| 2R9FA \| \| 2RA6A \| \| 2RA8A \| \| 2RA9A \| \| 2RB7A \| \| 2RB8A \| \| 2RBBA \| \| 2RBDA \| \| 2RBGA \| \| 2RBKA \| \| 2RC3A \| \| 2RDCA \| \| 2RDEA \| \| 2RDGA \| \| 2RDIA \| \| 2RDKA \| \| 2RDMA \| \| 2RDQA \| \| 2RDXA \| \| 2REEA \| \| 2RERA \| \| 2REUA \| \| 2RFFA \| \| 2RFQA \| \| 2RG4A \| \| 2RG8A \| \| 2RGIA \| \| 2RH0A \| \| 2RH2A \| | \| 2RH3A \| \| --- \| \| 2RHFA \| \| 2RIJA \| \| 2RILA \| \| 2RINA \| \| 2RIQA \| \| 2RIVA \| \| 2RIVB \| \| 2RJ2A \| \| 2RJIA \| \| 2RK3A \| \| 2RK9A \| \| 2RKLA \| \| 2RKNA \| \| 2RKQA \| \| 2RKVA \| \| 2RL8A \| \| 2RLDA \| \| 2SAKA \| \| 2SICI \| \| 2SPCA \| \| 2SQCA \| \| 2TGIA \| \| 2TNFA \| \| 2TPSA \| \| 2UUIA \| \| 2UURA \| \| 2UUUA \| \| 2UUYB \| \| 2UV4A \| \| 2UVJA \| \| 2UVKA \| \| 2UVOA \| \| 2UVPA \| \| 2UW1A \| \| 2UWAA \| \| 2UWJE \| \| 2UXQA \| \| 2UXYA \| \| 2UY1A \| \| 2UY2A \| \| 2UYOA \| \| 2UYTA \| \| 2UYZB \| | \| 2UZ1A \| \| --- \| \| 2UZCA \| \| 2V05A \| \| 2V0PA \| \| 2V1MA \| \| 2V1OA \| \| 2V1TA \| \| 2V26A \| \| 2V2GA \| \| 2V2PA \| \| 2V33A \| \| 2V3GA \| \| 2V3IA \| \| 2V3KA \| \| 2V3SA \| \| 2V4XA \| \| 2V6VA \| \| 2V6XA \| \| 2V6XB \| \| 2V75A \| \| 2V76A \| \| 2V79A \| \| 2V7FA \| \| 2V7KA \| \| 2V84A \| \| 2V89A \| \| 2V8HA \| \| 2V8IA \| \| 2V8TA \| \| 2V94A \| \| 2V9KA \| \| 2V9LA \| \| 2V9VA \| \| 2VB1A \| \| 2VBKA \| \| 2VBUA \| \| 2VC8A \| \| 2VCHA \| \| 2VCLA \| \| 2VDFA \| \| 2VDJA \| \| 2VE8A \| \| 2VEBA \| \| 2VECA \| | \| 2VESA \| \| --- \| \| 2VFKA \| \| 2VFOA \| \| 2VFRA \| \| 2VFXA \| \| 2VG0A \| \| 2VGOC \| \| 2VGXA \| \| 2VH3A \| \| 2VHAA \| \| 2VHBA \| \| 2VHJA \| \| 2VHKA \| \| 2VJIA \| \| 2VK2A \| \| 2VK8A \| \| 2VKJA \| \| 2VLGA \| \| 2VLIA \| \| 2VLQA \| \| 2VLQB \| \| 2VM9A \| \| 2VN6A \| \| 2VN6B \| \| 2VNGA \| \| 2VO4A \| \| 2VOKA \| \| 2VOVA \| \| 2VPAA \| \| 2VPBA \| \| 2VPNA \| \| 2VPTA \| \| 2VQ2A \| \| 2VQ4A \| \| 2VQGA \| \| 2VQPA \| \| 2VROA \| \| 2VRSA \| \| 2VRWB \| \| 2VS0A \| \| 2VSMA \| \| 2VSMB \| \| 2VTCA \| \| 2VTWA \| | \| 2VUWA \| \| --- \| \| 2VV6A \| \| 2VVEA \| \| 2VVPA \| \| 2VWSA \| \| 2VXGA \| \| 2VXNA \| \| 2VXQA \| \| 2VXTI \| \| 2VXZA \| \| 2VY8A \| \| 2VZCA \| \| 2VZPA \| \| 2W08A \| \| 2W0GA \| \| 2W15A \| \| 2W18A \| \| 2W1JA \| \| 2W1RA \| \| 2W1SA \| \| 2W1ZA \| \| 2W2EA \| \| 2W2RA \| \| 2W31A \| \| 2W39A \| \| 2W3GA \| \| 2W3PA \| \| 2W3QA \| \| 2W3XA \| \| 2W3YA \| \| 2W40A \| \| 2W50A \| \| 2W5EA \| \| 2W5NA \| \| 2W5QA \| \| 2W61A \| \| 2W6AA \| \| 2W6KA \| \| 2W7AA \| \| 2W7ZA \| \| 2W83C \| \| 2W86A \| \| 2W8TA \| \| 2W8XA \| | \| 2W91A \| \| --- \| \| 2W9YA \| \| 2WAGA \| \| 2WAOA \| \| 2WBFX \| \| 2WBMA \| \| 2WBNA \| \| 2WCJA \| \| 2WCRA \| \| 2WCWA \| \| 2WDCA \| \| 2WE3A \| \| 2WEWA \| \| 2WFIA \| \| 2WFOA \| \| 2WFPA \| \| 2WFWA \| \| 2WG7A \| \| 2WH7A \| \| 2WHLA \| \| 2WHMA \| \| 2WI8A \| \| 2WIYA \| \| 2WJ1A \| \| 2WJ5A \| \| 2WJEA \| \| 2WJNC \| \| 2WJRA \| \| 2WJWA \| \| 2WK1A \| \| 2WKJA \| \| 2WLGA \| \| 2WLUA \| \| 2WLVA \| \| 2WN9A \| \| 2WNFA \| \| 2WNPF \| \| 2WNXA \| \| 2WOLA \| \| 2WOZA \| \| 2WQIA \| \| 2WQKA \| \| 2WQPA \|   2WUUA |
| \| 2WUXA \| \| --- \| \| 2WVXA \| \| 2WWXB \| \| 2WYAA \| \| 2WYEA \| \| 2WYEB \| \| 2WZ1A \| \| 2WZGA \| \| 2YQCA \| \| 2YV4A \| \| 2YVIA \| \| 2YVQA \| \| 2YVRA \| \| 2YVSA \| \| 2YVTA \| \| 2YWIA \| \| 2YWWA \| \| 2YXNA \| \| 2YXOA \| \| 2YYKA \| \| 2YYOA \| \| 2YZHA \| \| 2YZSA \| \| 2YZTA \| \| 2YZYA \| \| 2Z08A \| \| 2Z0AA \| \| 2Z0BA \| \| 2Z0DA \| \| 2Z0JA \| \| 2Z0QA \| \| 2Z0TA \| \| 2Z0XA \| \| 2Z10A \| \| 2Z14A \| \| 2Z1CA \| \| 2Z1EA \| \| 2Z26A \| \| 2Z2NA \| \| 2Z30B \| \| 2Z3QA \| \| 2Z3QB \| \| 2Z51A \| \| 2Z5BA \| | \| 2Z5BB \| \| --- \| \| 2Z5EA \| \| 2Z5WA \| \| 2Z6FA \| \| 2Z6OA \| \| 2Z6RA \| \| 2Z72A \| \| 2Z7FI \| \| 2Z84A \| \| 2Z8FA \| \| 2Z8ZA \| \| 2Z98A \| \| 2Z9WA \| \| 2ZA0A \| \| 2ZA4B \| \| 2ZAYA \| \| 2ZB4A \| \| 2ZBCA \| \| 2ZBIA \| \| 2ZBLA \| \| 2ZCAA \| \| 2ZCMA \| \| 2ZCOA \| \| 2ZCWA \| \| 2ZD7A \| \| 2ZDHA \| \| 2ZDPA \| \| 2ZE3A \| \| 2ZEXA \| \| 2ZF9A \| \| 2ZFDA \| \| 2ZFDB \| \| 2ZFGA \| \| 2ZFYA \| \| 2ZFZA \| \| 2ZGIA \| \| 2ZGLA \| \| 2ZGYA \| \| 2ZHJA \| \| 2ZHNA \| \| 2ZHPA \| \| 2ZJ3A \| \| 2ZK9X \| \| 2ZKMX \| | \| 2ZKZA \| \| --- \| \| 2ZL7A \| \| 2ZNRA \| \| 2ZOGA \| \| 2ZOSA \| \| 2ZOUA \| \| 2ZPMA \| \| 2ZPTX \| \| 2ZPUA \| \| 2ZQ0A \| \| 2ZQ5A \| \| 2ZQEA \| \| 2ZQMA \| \| 2ZQOA \| \| 2ZS0D \| \| 2ZSIB \| \| 2ZSJA \| \| 2ZUVA \| \| 2ZUXA \| \| 2ZVYA \| \| 2ZW2A \| \| 2ZWAA \| \| 2ZWSA \| \| 2ZWUA \| \| 2ZX2A \| \| 2ZXYA \| \| 2ZYHA \| \| 2ZYJA \| \| 2ZYZB \| \| 2ZZDA \| \| 2ZZJA \| \| 2ZZVA \| \| 3A07A \| \| 3A0YA \| \| 3A1GA \| \| 3A1GB \| \| 3A35A \| \| 3A39A \| \| 3A4CA \| \| 3A9IA \| \| 3B2YA \| \| 3B33A \| \| 3B34A \| \| 3B44A \| | \| 3B47A \| \| --- \| \| 3B49A \| \| 3B4NA \| \| 3B4QA \| \| 3B4UA \| \| 3B50A \| \| 3B5EA \| \| 3B5MA \| \| 3B5OA \| \| 3B64A \| \| 3B6EA \| \| 3B6HA \| \| 3B79A \| \| 3B7CA \| \| 3B7LA \| \| 3B7SA \| \| 3B8FA \| \| 3B9OA \| \| 3B9TA \| \| 3B9WA \| \| 3BA1A \| \| 3BA3A \| \| 3BB0A \| \| 3BB7A \| \| 3BBBA \| \| 3BBYA \| \| 3BC1B \| \| 3BC9A \| \| 3BCWA \| \| 3BCYA \| \| 3BDUA \| \| 3BDVA \| \| 3BE6A \| \| 3BEDA \| \| 3BEIA \| \| 3BEMA \| \| 3BEOA \| \| 3BEXA \| \| 3BF7A \| \| 3BFMA \| \| 3BFOA \| \| 3BFQG \| \| 3BFVA \| \| 3BGEA \| | \| 3BGYA \| \| --- \| \| 3BHDA \| \| 3BHNA \| \| 3BHOA \| \| 3BHWA \| \| 3BHYA \| \| 3BI1A \| \| 3BI7A \| \| 3BIOA \| \| 3BIQA \| \| 3BIYA \| \| 3BJ4A \| \| 3BJDA \| \| 3BJEA \| \| 3BJNA \| \| 3BK5A \| \| 3BKXA \| \| 3BL9A \| \| 3BLDA \| \| 3BLZA \| \| 3BM1A \| \| 3BM4A \| \| 3BMXA \| \| 3BMZA \| \| 3BN0A \| \| 3BNEA \| \| 3BNYA \| \| 3BO6A \| \| 3BODA \| \| 3BOEA \| \| 3BOFA \| \| 3BOGA \| \| 3BONA \| \| 3BP1A \| \| 3BP6A \| \| 3BP6B \| \| 3BPJA \| \| 3BPKA \| \| 3BPTA \| \| 3BPVA \| \| 3BPWA \| \| 3BQ9A \| \| 3BQAA \| \| 3BQKA \| | \| 3BQOA \| \| --- \| \| 3BQPA \| \| 3BQXA \| \| 3BRCA \| \| 3BS2A \| \| 3BS3A \| \| 3BS4A \| \| 3BS6A \| \| 3BT5A \| \| 3BUTA \| \| 3BUUA \| \| 3BUXB \| \| 3BV8A \| \| 3BVFA \| \| 3BVXA \| \| 3BWHA \| \| 3BWLA \| \| 3BWSA \| \| 3BWUD \| \| 3BWUF \| \| 3BWXA \| \| 3BWZA \| \| 3BXPA \| \| 3BXUA \| \| 3BY4A \| \| 3BY8A \| \| 3BY9A \| \| 3BYPA \| \| 3BYQA \| \| 3BZWA \| \| 3C0FB \| \| 3C18A \| \| 3C1AA \| \| 3C1QA \| \| 3C2EA \| \| 3C2QA \| \| 3C2UA \| \| 3C37A \| \| 3C4BA \| \| 3C4SA \| \| 3C57A \| \| 3C5KA \| \| 3C5NA \| \| 3C68A \| | \| 3C6AA \| \| --- \| \| 3C6KA \| \| 3C6WA \| \| 3C70A \| \| 3C7MA \| \| 3C7TA \| \| 3C7XA \| \| 3C8CA \| \| 3C8EA \| \| 3C8IA \| \| 3C8LA \| \| 3C8MA \| \| 3C8NA \| \| 3C8WA \| \| 3C8ZA \| \| 3C9AA \| \| 3C9FA \| \| 3C9HA \| \| 3C9IA \| \| 3C9PA \| \| 3C9QA \| \| 3C9UA \| \| 3CA7A \| \| 3CA8A \| \| 3CANA \| \| 3CBNA \| \| 3CBPA \| \| 3CBWA \| \| 3CBZA \| \| 3CCDA \| \| 3CECA \| \| 3CETA \| \| 3CEXA \| \| 3CG6A \| \| 3CGIA \| \| 3CGXA \| \| 3CH4B \| \| 3CHBD \| \| 3CHHA \| \| 3CHJA \| \| 3CHMA \| \| 3CHVA \| \| 3CI3A \| \| 3CI6A \| |
| \| 3CI9A \| \| --- \| \| 3CIJA \| \| 3CIMA \| \| 3CINA \| \| 3CITA \| \| 3CJ1A \| \| 3CJDA \| \| 3CJKB \| \| 3CJPA \| \| 3CJSA \| \| 3CJSB \| \| 3CJYA \| \| 3CK6A \| \| 3CKCA \| \| 3CKJA \| \| 3CKKA \| \| 3CKMA \| \| 3CL5A \| \| 3CL6A \| \| 3CLAA \| \| 3CLMA \| \| 3CM3A \| \| 3CMGA \| \| 3CNBA \| \| 3CNEA \| \| 3CNUA \| \| 3CNVA \| \| 3CNYA \| \| 3COVA \| \| 3CP0A \| \| 3CP3A \| \| 3CP7A \| \| 3CPGA \| \| 3CPQA \| \| 3CPTA \| \| 3CPTB \| \| 3CQ1A \| \| 3CQDA \| \| 3CQLA \| \| 3CSXA \| \| 3CT5A \| \| 3CT6A \| \| 3CTPA \| \| 3CTZA \| | \| 3CU2A \| \| --- \| \| 3CU9A \| \| 3CUZA \| \| 3CVEA \| \| 3CVJA \| \| 3CVOA \| \| 3CW9A \| \| 3CWNA \| \| 3CWRA \| \| 3CWVA \| \| 3CWWA \| \| 3CX5A \| \| 3CX5B \| \| 3CX5C \| \| 3CX5D \| \| 3CX5F \| \| 3CX5G \| \| 3CX5H \| \| 3CX5I \| \| 3CXNA \| \| 3CYPB \| \| 3CZ1A \| \| 3CZ6A \| \| 3CZPA \| \| 3CZXA \| \| 3D00A \| \| 3D02A \| \| 3D06A \| \| 3D0FA \| \| 3D0JA \| \| 3D0KA \| \| 3D0WA \| \| 3D1BA \| \| 3D1KA \| \| 3D1MA \| \| 3D1PA \| \| 3D1RA \| \| 3D2QA \| \| 3D2YA \| \| 3D30A \| \| 3D32A \| \| 3D33A \| \| 3D34A \| \| 3D3BA \| | \| 3D3BJ \| \| --- \| \| 3D3MA \| \| 3D3YA \| \| 3D47A \| \| 3D4EA \| \| 3D4UB \| \| 3D59A \| \| 3D6IA \| \| 3D6KA \| \| 3D6RA \| \| 3D7AA \| \| 3D7IA \| \| 3D7JA \| \| 3D85C \| \| 3D9NA \| \| 3D9XA \| \| 3DA0A \| \| 3DA4A \| \| 3DA5A \| \| 3DAAA \| \| 3DALA \| \| 3DANA \| \| 3DB2A \| \| 3DB7A \| \| 3DBOA \| \| 3DCDA \| \| 3DCMX \| \| 3DCZA \| \| 3DD7A \| \| 3DDCB \| \| 3DEFA \| \| 3DFFA \| \| 3DFGA \| \| 3DG6A \| \| 3DG9A \| \| 3DGPA \| \| 3DGPB \| \| 3DHAA \| \| 3DHFA \| \| 3DHUA \| \| 3DI4A \| \| 3DJEA \| \| 3DJHA \| \| 3DK9A \| | \| 3DKSA \| \| --- \| \| 3DLCA \| \| 3DLUA \| \| 3DMLA \| \| 3DMNA \| \| 3DN7A \| \| 3DNFA \| \| 3DNHA \| \| 3DNJA \| \| 3DNUA \| \| 3DNXA \| \| 3DNZA \| \| 3DO6A \| \| 3DOUA \| \| 3DQGA \| \| 3DQYA \| \| 3DR2A \| \| 3DR4A \| \| 3DR9A \| \| 3DRFA \| \| 3DRWA \| \| 3DS4A \| \| 3DSBA \| \| 3DSHA \| \| 3DSMA \| \| 3DSOA \| \| 3DSSA \| \| 3DSZA \| \| 3DT5A \| \| 3DTBA \| \| 3DTZA \| \| 3DUPA \| \| 3DXEA \| \| 3DXLA \| \| 3DXTA \| \| 3DYJA \| \| 3DZ1A \| \| 3DZAA \| \| 3DZZA \| \| 3E03A \| \| 3E05A \| \| 3E0EA \| \| 3E0XA \| \| 3E0ZA \| | \| 3E11A \| \| --- \| \| 3E19A \| \| 3E1EA \| \| 3E1RA \| \| 3E21A \| \| 3E2DA \| \| 3E2OA \| \| 3E2QA \| \| 3E2VA \| \| 3E3UA \| \| 3E3XA \| \| 3E48A \| \| 3E4GA \| \| 3E4VA \| \| 3E4WA \| \| 3E5UA \| \| 3E61A \| \| 3E7DA \| \| 3E7HA \| \| 3E7RL \| \| 3E86A \| \| 3E8OA \| \| 3E8TA \| \| 3E96A \| \| 3E9KA \| \| 3E9VA \| \| 3EA1A \| \| 3EA6A \| \| 3EAFA \| \| 3EBKA \| \| 3EBQA \| \| 3EBTA \| \| 3EBVA \| \| 3EC3A \| \| 3EC4A \| \| 3ECBB \| \| 3ECFA \| \| 3EDHA \| \| 3EDOA \| \| 3EDVA \| \| 3EE4A \| \| 3EEAA \| \| 3EEHA \| \| 3EEIA \| | \| 3EERA \| \| --- \| \| 3EF8A \| \| 3EFGA \| \| 3EFYA \| \| 3EGAA \| \| 3EH1A \| \| 3EHMA \| \| 3EI9A \| \| 3EIKA \| \| 3EINA \| \| 3EIPA \| \| 3EJ9A \| \| 3EJ9B \| \| 3EJKA \| \| 3EJNA \| \| 3EKIA \| \| 3EL6A \| \| 3ELFA \| \| 3ELJA \| \| 3ELKA \| \| 3ELLA \| \| 3ELNA \| \| 3ELQA \| \| 3EMFA \| \| 3EMHA \| \| 3EMIA \| \| 3EMRA \| \| 3EN8A \| \| 3ENBA \| \| 3ENUA \| \| 3EO5A \| \| 3EO6A \| \| 3EO7A \| \| 3EOIA \| \| 3EOJA \| \| 3EPVA \| \| 3EPWA \| \| 3EQNA \| \| 3EQXA \| \| 3ER6A \| \| 3ER7A \| \| 3ES4A \| \| 3ESLA \| \| 3ESMA \| | \| 3ESSA \| \| --- \| \| 3ETJA \| \| 3ETOA \| \| 3ETVA \| \| 3ETZA \| \| 3EURA \| \| 3EVFA \| \| 3EVYA \| \| 3EW0A \| \| 3EW8A \| \| 3EWNA \| \| 3EWYA \| \| 3EYEA \| \| 3EYTA \| \| 3EZIA \| \| 3F0DA \| \| 3F0HA \| \| 3F0PA \| \| 3F1LA \| \| 3F1PB \| \| 3F2EA \| \| 3F2KA \| \| 3F2ZA \| \| 3F3KA \| \| 3F40A \| \| 3F43A \| \| 3F47A \| \| 3F4MA \| \| 3F4SA \| \| 3F59A \| \| 3F5HA \| \| 3F62A \| \| 3F67A \| \| 3F6CA \| \| 3F6GA \| \| 3F6KA \| \| 3F6VA \| \| 3F6YA \| \| 3F75P \| \| 3F7EA \| \| 3F7LA \| \| 3F7QA \| \| 3F7WA \| \| 3F8MA \| |
| \| 3F8TA \| \| --- \| \| 3F8XA \| \| 3F95A \| \| 3F9MA \| \| 3F9SA \| \| 3F9XA \| \| 3FAPB \| \| 3FAUA \| \| 3FB9A \| \| 3FBLA \| \| 3FCIA \| \| 3FCNA \| \| 3FD3A \| \| 3FD5A \| \| 3FDGA \| \| 3FDHA \| \| 3FDJA \| \| 3FEAA \| \| 3FEGA \| \| 3FF1A \| \| 3FF2A \| \| 3FF5A \| \| 3FF9A \| \| 3FFDP \| \| 3FFRA \| \| 3FFVA \| \| 3FFYA \| \| 3FG8A \| \| 3FG9A \| \| 3FGHA \| \| 3FGRA \| \| 3FGRB \| \| 3FGVA \| \| 3FH1A \| \| 3FH2A \| \| 3FHDA \| \| 3FHFA \| \| 3FHLA \| \| 3FHVA \| \| 3FIAA \| \| 3FIDA \| \| 3FILA \| \| 3FJ1A \| \| 3FJUB \| | \| 3FJVA \| \| --- \| \| 3FK8A \| \| 3FKAA \| \| 3FKCA \| \| 3FKEA \| \| 3FL2A \| \| 3FM2A \| \| 3FMCA \| \| 3FMUA \| \| 3FN4A \| \| 3FN5A \| \| 3FO3A \| \| 3FO5A \| \| 3FO8D \| \| 3FOJA \| \| 3FOTA \| \| 3FOVA \| \| 3FPNA \| \| 3FPNB \| \| 3FPWA \| \| 3FQGA \| \| 3FQMA \| \| 3FR7A \| \| 3FRHA \| \| 3FRQA \| \| 3FS8A \| \| 3FSAA \| \| 3FSOA \| \| 3FSSA \| \| 3FSTA \| \| 3FTJA \| \| 3FUCA \| \| 3FUTA \| \| 3FVHA \| \| 3FVSA \| \| 3FVYA \| \| 3FW9A \| \| 3FWKA \| \| 3FWNA \| \| 3FWYA \| \| 3FWZA \| \| 3FX7A \| \| 3FYBA \| \| 3FYRA \| | \| 3FZ9A \| \| --- \| \| 3FZEA \| \| 3G02A \| \| 3G0MA \| \| 3G0OA \| \| 3G16A \| \| 3G1AA \| \| 3G1JA \| \| 3G1PA \| \| 3G1ZA \| \| 3G21A \| \| 3G23A \| \| 3G2BA \| \| 3G2EA \| \| 3G36A \| \| 3G3KA \| \| 3G3SA \| \| 3G3TA \| \| 3G40A \| \| 3G46A \| \| 3G48A \| \| 3G5BA \| \| 3G5SA \| \| 3G5TA \| \| 3G63A \| \| 3G7GA \| \| 3G7PA \| \| 3G7RA \| \| 3G7UA \| \| 3G85A \| \| 3G89A \| \| 3G8YA \| \| 3G98A \| \| 3G9RA \| \| 3GA3A \| \| 3GA4A \| \| 3GAEA \| \| 3GAZA \| \| 3GB5A \| \| 3GBWA \| \| 3GBYA \| \| 3GD0A \| \| 3GD6A \| \| 3GDWA \| | \| 3GE3A \| \| --- \| \| 3GE3C \| \| 3GE3E \| \| 3GF3A \| \| 3GF6A \| \| 3GFAA \| \| 3GFPA \| \| 3GFVA \| \| 3GG4A \| \| 3GG7A \| \| 3GGNA \| \| 3GGQA \| \| 3GGYA \| \| 3GHAA \| \| 3GI7A \| \| 3GIUA \| \| 3GIWA \| \| 3GIYA \| \| 3GJ8B \| \| 3GJYA \| \| 3GK6A \| \| 3GK7A \| \| 3GKJA \| \| 3GKNA \| \| 3GMGA \| \| 3GMIA \| \| 3GMXA \| \| 3GN6A \| \| 3GNEA \| \| 3GNLA \| \| 3GNZP \| \| 3GO5A \| \| 3GO9A \| \| 3GOCA \| \| 3GOEA \| \| 3GOHA \| \| 3GONA \| \| 3GP4A \| \| 3GPGA \| \| 3GQHA \| \| 3GQQA \| \| 3GR3A \| \| 3GR4A \| \| 3GREA \| | \| 3GRLA \| \| --- \| \| 3GS9A \| \| 3GVEA \| \| 3GVOA \| \| 3GWBA \| \| 3GWIA \| \| 3GWNA \| \| 3GWQA \| \| 3GXWA \| \| 3GY9A \| \| 3GYBA \| \| 3GYCA \| \| 3GYKA \| \| 3GZAA \| \| 3GZBA \| \| 3GZDA \| \| 3GZRA \| \| 3H05A \| \| 3H09A \| \| 3H0NA \| \| 3H0UA \| \| 3H12A \| \| 3H1DA \| \| 3H20A \| \| 3H2ZA \| \| 3H36A \| \| 3H3LA \| \| 3H4TA \| \| 3H51A \| \| 3H5LA \| \| 3H63A \| \| 3H6JA \| \| 3H6PA \| \| 3H6PC \| \| 3H6QA \| \| 3H6RA \| \| 3H74A \| \| 3H75A \| \| 3H79A \| \| 3H7CX \| \| 3H7HB \| \| 3H87A \| \| 3H87C \| \| 3H8HA \| | \| 3H8TA \| \| --- \| \| 3H8ZA \| \| 3H9CA \| \| 3H9MA \| \| 3H9WA \| \| 3HA2A \| \| 3HBMA \| \| 3HC1A \| \| 3HC7A \| \| 3HCJA \| \| 3HDOA \| \| 3HFOA \| \| 3HFTA \| \| 3HGLA \| \| 3HH1A \| \| 3HHSA \| \| 3HHTA \| \| 3HHTB \| \| 3HIDA \| \| 3HIUA \| \| 3HL1A \| \| 3HLFA \| \| 3HLZA \| \| 3HM4A \| \| 3HM5A \| \| 3HN0A \| \| 3HN5A \| \| 3HNOA \| \| 3HO6A \| \| 3HOIA \| \| 3HOLA \| \| 3HP4A \| \| 3HPCX \| \| 3HR0A \| \| 3HR6A \| \| 3HR9A \| \| 3HRGA \| \| 3HRLA \| \| 3HROA \| \| 3HRQA \| \| 3HSAA \| \| 3HSHA \| \| 3HSRA \| \| 3HT1A \| | \| 3HTUA \| \| --- \| \| 3HTYA \| \| 3HUPA \| \| 3HUUA \| \| 3HV8A \| \| 3HVIA \| \| 3HVWA \| \| 3HW5A \| \| 3HWPA \| \| 3HWUA \| \| 3HWWA \| \| 3HX8A \| \| 3HX9A \| \| 3HXJA \| \| 3HXLA \| \| 3HY0A \| \| 3HYNA \| \| 3HZ7A \| \| 3HZ8A \| \| 3HZPA \| \| 3I06A \| \| 3I09A \| \| 3I0ZA \| \| 3I24A \| \| 3I2VA \| \| 3I2ZA \| \| 3I31A \| \| 3I3QA \| \| 3I45A \| \| 3I4GA \| \| 3I4ZA \| \| 3I57A \| \| 3I7MA \| \| 3I84A \| \| 3I94A \| \| 3IARA \| \| 3IB5A \| \| 3IB7A \| \| 3IBWA \| \| 3IC3A \| \| 3IC4A \| \| 3ICVA \| \| 3ID1A \| \| 3IDBB \| |
| \| 3IDFA \| \| --- \| \| 3IDUA \| \| 3IE4A \| \| 3IE7A \| \| 3IEEA \| \| 3IFTA \| \| 3IG9A \| \| 3IGFA \| \| 3IGNA \| \| 3IGRA \| \| 3IGSA \| \| 3IHTA \| \| 3IHVA \| \| 3IISM \| \| 3IIXA \| \| 3IJ6A \| \| 3IJDA \| \| 3IJMA \| \| 3IJWA \| \| 3IKBA \| \| 3KK4A \| | \| 3IUOA \| \| --- \| \| 3IUWA \| \| 3IUZA \| \| 3IV0A \| \| 3IV4A \| \| 3IVVA \| \| 3IWFA \| \| 3IX3A \| \| 3JQ0A \| \| 3JQ1A \| \| 3JRNA \| \| 3JRVA \| \| 3JSLA \| \| 3JSRA \| \| 3JTFA \| \| 3JTWA \| \| 3JTXA \| \| 3JTZA \| \| 3JUIA \| \| 3JUMA \| \| 3JVLA \| | \| 3KC2A \| \| --- \| \| 3KD4A \| \| 3KD6A \| \| 3KDFA \| \| 3KDFB \| \| 3KDHA \| \| 3KDWA \| \| 3KE7A \| \| 3KEBA \| \| 3KEPA \| \| 3KEVA \| \| 3KEZA \| \| 3KF6A \| \| 3KF6B \| \| 3KG4A \| \| 3KGWA \| \| 3KGYA \| \| 3KGZA \| \| 3KH1A \| \| 3KHIA \| \| 3KIZA \| | \| 3PCGA \| \| --- \| \| 3PROC \| \| 3THIA \| \| 3TSSA \| \| 3VUBA \| \| 4MT2A \| \| 4UBPA \| \| 4UBPB \| \| 4UBPC \| \| 7A3HA \| \| 7AHLA \| \| 7ODCA \| \| 8ABPA \| \| 3IKWA \| \| 3ILVA \| \| 3ILWA \| \| 3ILXA \| \| 3IM6A \| \| 3IMKA \| \| 3IMMA \| \| 3JX9A \| | \| 3IP0A \| \| --- \| \| 3IP4C \| \| 3IPFA \| \| 3IPJA \| \| 3IR4A \| \| 3IRBA \| \| 3IS6A \| \| 3ISQA \| \| 3ISRA \| \| 3ISXA \| \| 3IT3A \| \| 3ITEA \| \| 3ITQA \| \| 3IUFA \| \| 3IUKA \| \| 3JXOA \| \| 3JXSA \| \| 3JY6A \| \| 3JYZA \| \| 3JZ0A \| \| 3K0ZA \| | \| 3K1TA \| \| --- \| \| 3K1UA \| \| 3K26A \| \| 3K29A \| \| 3K2OA \| \| 3K4IA \| \| 3K67A \| \| 3K69A \| \| 3K6OA \| \| 3K6QA \| \| 3K7CA \| \| 3K7XA \| \| 3K9DA \| \| 3KA5A \| \| 3KBGA \| \| 3KKGA \| \| 3KKIA \| \| 3KMIA \| \| 3KOGA \| \| 3KOJA \|   3K12A | \| 3KPEA \| \| --- \| \| 3KQ5A \| \| 3KR7A \| \| 3KS6A \| \| 3KS9A \| \| 3KSTA \| \| 3KTCA \| \| 3KTFA \| \| 3KTOA \| \| 3KUPA \| \| 3KV1A \| \| 3KWRA \| \| 3KWSA \| \| 3KYAA \| \| 3KZPA \| \| 3INOA \| \| 3IO3A \| \| 3KKBA \| \| 3KKFA \| |
