# Supplementary material for: Predicting Turns in Proteins with a Unified Model
Source: PLoS One. 2012 Nov 7;7(11):e48389. doi: 10.1371/journal.pone.0048389 (PMC3492357; doi:10.1371/journal.pone.0048389)
Supplement: Table S2 — Detailed results of datasets. (DOCX) [file pone.0048389.s006.docx]

Table S2. Detailed results of datasets

|  | Ac(%) | Q_pred_ (%) | Sn(%) | Sp(%) | MCC | Sw(%) | AUC |
| --- | --- | --- | --- | --- | --- | --- | --- |
| Train_0925 5-fold validation | 88.8 | 79.9 | 71.8 | 94.2 | 0.69 | 66.0 | 0.96 |
| Test_1025 | 82.6 | 64.4 | 60.8 | 89.5 | 0.51 | 50.3 | 0.87 |
| EVAset1 | 79.6 | 62.5 | 49.0 | 90.0 | 0.43 | 39.0 | 0.82 |
| CASP9 | 79.3 | 55.5 | 54.1 | 86.9 | 0.41 | 41.0 | 0.82 |
