# Supplementary material for: Predicting Turns in Proteins with a Unified Model
Source: PLoS One. 2012 Nov 7;7(11):e48389. doi: 10.1371/journal.pone.0048389 (PMC3492357; doi:10.1371/journal.pone.0048389)
Supplement: Text S1 — PDB ID list of Train_0925. (DOCX) [file pone.0048389.s007.docx]

## Support information-Text S1

**S1. PDB ID list of Train_0925**

PDB IDs of 4107 sequences in the Train_0925 are listed as below.
