# Supplementary material for: Predicting Turns in Proteins with a Unified Model
Source: PLoS One. 2012 Nov 7;7(11):e48389. doi: 10.1371/journal.pone.0048389 (PMC3492357; doi:10.1371/journal.pone.0048389)
Supplement: Text S2 — Details of results. (DOCX) [file pone.0048389.s008.docx]

## Support information-Text S2

**S2. Details of results**

We collected results of all the datasets we used in a combinational table (Table S2). We also calculated the Sw and AUC of them. Sw is a commonly used measure in other structure prediction field, such as disorder prediction [1], it can be calculated as Sn +Sp -1. It is more comprehensive evaluation, and the higher it is, the better the prediction. The area under the ROC curve (AUC) is a reasonable measure to assess a predictor. The value of AUC is between 0 and 1, the bigger the value is, the better the prediction.

**References:**

1. Zhang T, Faraggi E, Xue B, Dunker AK, Uversky VN, et al. (2012) SPINE-D: accurate prediction of short and long disordered regions by a single neural-network based method. J Biomol Struct Dyn 29: 799-813.
