# Supplementary material for: Predicting Turns in Proteins with a Unified Model
Source: PLoS One. 2012 Nov 7;7(11):e48389. doi: 10.1371/journal.pone.0048389 (PMC3492357; doi:10.1371/journal.pone.0048389)
Supplement: Text S3 — Secondary structure prediction-SPSSMPred. (DOCX) [file pone.0048389.s009.docx]

## Support information-Text S3

**S3. Secondary structure prediction-SPSSMPred**

SPSSMPred is an accurate predictor of protein secondary structure [1]. The innovative technologies are (i) an original structural position-specific scoring matrix (SPSSM), (ii) the 9M_database, and (iii) profile encodings.

**S3.1 SPSSM**

The SPSSM is a distinctive PSSM-like profile composed from three boxes, where the SPSSM scores are used to appraise matched sequences after alignments are stored (Figure S1). The score is defined as

$Score1\left(i,s\right)=\sum_{A(i,j)} \theta\left(S\left(i,j\right),s\right)$ (1)

where *i* is the position of an amino acid in the target sequence and *s* is one of the three state secondary structure elements, H, E or C. *A*(*i,j*) denotes a set of all the matched sequence’s amino acids at the position *i*, and *j* directs matched sequences in *A*(*i,j*). *S*(*i,j*) represents the corresponding secondary structure element of *A*(*i,j*). *θ*(*S*(*i,j*),s) is defined as

$\theta\left(S\left(i,j\right),s\right)=\left\{\begin{matrix} 1 \\ 0 \end{matrix}\right.\begin{matrix} if \\ else \end{matrix}\begin{matrix} S\left(i,j\right)=s \\ \end{matrix}$ (2)

The scores for the state of secondary structures, H, E and C, are calculated separately, then allotted to the corresponding three boxes. It is clear that the score is the frequency of the secondary structural elements, and that there are three elements for each amino acid in the sequences.

There are two forms of SPSSM; the raw SPSSM and the normalized SPSSM. The former is mainly stored in the 9M_database (see below), and the latter is one of the results produced by BLAST.

The raw SPSSM in the 9M_database is obtained by first running PSI-BLAST against the PDB_99 (with three iterations and 500 maximum target sequences) to find homologous sequences relative to the target sequence (Figure S1). PDB_99 is constructed by using CDHIT for single-copy sequences, with sequence identity cut off value at 99% against sequences stored in PDB (as of 2010, containing 70 177 proteins) with a resolution of <2.5Å and an R-value of <0.3, and using only X-ray structure. The matched piecewise local sequences are then selected according to e-values that are below a given threshold (say 10). All the selected sequences are subsequently identified and ranked in accordance by their e-values in ascending order, and the top N (default is 10) of these sorted sequences that are considered as containing rich homologous information are reserved (if the number of the selected sequences were less than N, all the selected sequences would be kept). The scores of all amino acids in the 9M_database are then calculated according to Equation (1), and the final raw SPSSM is constructed.

When BLAST is executed for a query against the 9M_database, the procedure is similar to that mentioned above. However, there are two differences; one is that BLAST uses the raw SPSSM instead of the secondary structural elements, and the other is that the output of 9M-BLAST is the normalized SPSSM, which is defined as

$Score2\left(i,s\right)=\sum_{A(i,j)} P(i,j,s)/\sum_{S\in\left\{H,E,C\right\}} \sum_{A\left(i,j\right)} P\left(i,j,s\right)$ (3)

where *P*(*i,j,s*) denotes the value of the corresponding secondary structural profile set of *A*(*i,j*) in the 9M_database, where the raw SPSSM has been calculated according to Equation (1).

**S3.2 The 9M_database**

The 9M_database is a BLAST compatible database and is the kernel of the SPSSMPred, in which there are an unprecedented 9 million sequences and corresponding secondary structural profiles. The sequences in the 9M_database were derived from the non-redundant NCBI database (as of 2009, 9 069 431 proteins) applied in PSIBLAST. The secondary structural profiles of the 9M_database were generated by alignment and score [Equation (1)]. Each of the 9 million sequences was aligned against PDB_99 with PSIBLAST by setting the e-value at four different levels (1e-5, 1e-3, 1e-1 and 10) and other parameters at default. The aligned sequence segments and corresponding unions in PDB_99 were obtained in this way. The secondary structure elements in the matched unions were scored in three boxes that contained the scores of three state secondary structural elements. These boxes then constituted a secondary structural profile of the original sequence. This procedure was repeated until the profiles of all sequences in the 9M_database were formed.

In constructing the 9M_database, we have created a vast database with integrated sequences and secondary structural profiles. The 9M_database is based on the concept that local similarities in protein sequences typically exhibit conserved structures and also, in addition, that a high degree of robustness of the structure with respect to the sequence variation may represent a remote homology nature in the sequence.

**S3.3 Profile encodings**

We use widely applied sequence profiles as encodings for our predictor, as well as newly proposed structural profiles. Sequence profiles contain rich sequence evolution information, and have long been proved to be an effective variable for the prediction of secondary structures. On the other hand, structural profiles are very simple, include valuable structural evolution information derived from all the known detected structures and are evidently of significant importance in improving prediction performances. As a result, we utilize 23 variables [PSSM (20 variables) and SPSSM (3 variables). The clarified 23 variables are also shown in Fig. 1.] as our final total encodings, and establish a new relationship between structural profiles and secondary structures in modeling. In the case that the structural profile is not sufficient for encoding, the sequence profile will then take the dominant role in the prediction.

The flowchart of SPSSMPred and predicted results can be found in reference [2].

**References:**

1. Li D, Li T, Cong P, Xiong W, Sun J (2012) A novel structural position-specific scoring matrix for the prediction of protein secondary structures. Bioinformatics 28: 32-39.

2. Sun J, Tang S, Xiong W, Cong P, Li T (2012) DSP: a protein shape string and its profile prediction server. Nucleic Acids Res.
