# Supplementary material for: Predicting Turns in Proteins with a Unified Model
Source: PLoS One. 2012 Nov 7;7(11):e48389. doi: 10.1371/journal.pone.0048389 (PMC3492357; doi:10.1371/journal.pone.0048389)
Supplement: Text S5 — Webserver introduction. (DOCX) [file pone.0048389.s011.docx]

## Support information-Text S5

**S5. Webserver introduction**

There are two parts of our webserver (<http://cal.tongji.edu.cn/TurnP/index.jsp>): analysis part and prediction part. In the analysis part, users are allowed to search and display the turns of proteins among the TurnDB_09 online via submitting a PDB ID or download the entire database in a text format, and the output example is shown in Figure S3: turn, secondary structure, and a string diagram are displayed simultaneously.

In the prediction part, TurnP provides a powerful predictor of all type turns for a query or a file (FASTA format). An ID is demanded to mark each entry, and a random ID can be given while user submits only one query without ID. As shown in Figure S4, the output of a query sequence consists of predicted turns, predicted three-state secondary structure elements, predicted shape strings and the degree of probability (from 1 to 9) of each amino acid. The probability of turn and non-turn are identified in different color, for discriminating easily. To visualize the result, we created a concise and vivid colorful string as the secondary structure diagram, which can display even in low software requirement.
